# A blueprint for a multi-disease, multi-domain Bayesian adaptive platform trial incorporating adult and paediatric subgroups: the *Staphylococcus aureus* Network Adaptive Platform trial

**DOI:** 10.1186/s13063-023-07718-x

**Published:** 2023-12-06

**Authors:** Robert K. Mahar, Anna McGlothlin, Michael Dymock, Todd C. Lee, Roger J. Lewis, Thomas Lumley, Jocelyn Mora, David J. Price, Benjamin R. Saville, Tom Snelling, Rebecca Turner, Steven A. Webb, Joshua S. Davis, Steven Y. C. Tong, Julie A. Marsh, on behalf of the SNAP Global Trial Steering Committee, Asha Bowen, Matthew Cheng, Nick Daneman, Joshua Davis, Anna Goodman, George Heriot, Todd C. Lee, Roger Lewis, David Lye, Julie Marsh, Anna McGlothlin, Zoe McQuilten, Jocelyn Mora, Susan Morpeth, David Paterson, David Price, Jason Roberts, Owen Robinson, Matthew Scarborough, Steven Tong, Sebastiaan van Hal, Genevieve Walls, Steve Webb, Lynda Whiteway, Dafna Yahav

**Affiliations:** 1 Centre for Epidemiology and Biostatistics, Melbourne School of Population and Global Health, University of Melbourne, Parkville, Victoria Australia; 2 Clinical Epidemiology and Biostatistics Unit, Murdoch Children’s Research Institute, Parkville, Victoria Australia; 3 Centre for Data Science, Queensland University of Technology, Brisbane, Queensland Australia; 4 Berry Consultants LLC, Austin, Texas USA; 5 Wesfarmers Centre of Vaccines and Infectious Diseases, Telethon Kids Institute, Nedlands, Western Australia Australia; 6 Division of Infectious Diseases, Department of Medicine, McGill University, Montreal, Canada; 7 Department of Emergency Medicine, Harbor-UCLA Medical Center, Torrance, California USA; 8 Department of Statistics, University of Auckland, Auckland, New Zealand; 9 Department of Infectious Diseases, University of Melbourne at the Peter Doherty Institute for Infection and Immunity, Parkville, Victoria Australia; 10 Department of Biostatistics, Vanderbilt University Medical Center, Nashville, Tennessee USA; 11 Department of Infectious Diseases, Perth Children’s Hospital, Perth, Western Australia Australia; 12 Sydney School of Public Health, Faculty of Medicine and Health, University of Sydney, Sydney, New South Wales Australia; 13 Medical Research Council Clinical Trials Unit at University College London, London, United Kingdom; 14 St John of God Healthcare, Perth, Western Australia Australia; 15 Australian and New Zealand Intensive Care Research Centre, School of Public Health and Preventive Medicine, Monash University, Melbourne, Australia; 16 Department of Infectious Diseases, John Hunter Hospital, Newcastle, New South Wales Australia; 17 Menzies School of Health Research, Royal Darwin Hospital, Darwin, Northern Territory Australia; 18 Victorian Infectious Diseases Service, Royal Melbourne Hospital at the Peter Doherty Institute for Infection and Immunity, Melbourne, Victoria Australia

**Keywords:** Bayesian, Platform, *Staphylococcus aureus*, Trial, Randomised, Adaptive

## Abstract

The *Staphylococcus aureus* Network Adaptive Platform (SNAP) trial is a multifactorial Bayesian adaptive platform trial that aims to improve the way that *S. aureus* bloodstream infection, a globally common and severe infectious disease, is treated. In a world first, the SNAP trial will simultaneously investigate the effects of multiple intervention modalities within multiple groups of participants with different forms of *S. aureus* bloodstream infection. Here, we formalise the trial structure, modelling approach, and decision rules that will be used for the SNAP trial. By summarising the statistical principles governing the design, our hope is that the SNAP trial will serve as an adaptable template that can be used to improve comparative effectiveness research efficiency in other disease areas.

**Trial registration** NCT05137119. Registered on 30 November 2021.

**Supplementary Information:**

The online version contains supplementary material available at 10.1186/s13063-023-07718-x.

## Introduction

*Staphylococcus aureus* is the leading bacterial cause of global mortality overall, with more than 1 million estimated *S. aureus-*related deaths per year, and the leading bacterial cause of mortality due to bloodstream infections [1]. The 90-day all-cause mortality rate for *S. aureus* bloodstream infection, when treated with the best known therapies, is between 15 and 30% in developed countries [2].

The standard treatment for *S. aureus* bloodstream infection, antibiotics delivered intravenously for between 2 and 6 weeks, depends on the characteristics of the infecting *S. aureus* bacterium. Both methicillin-susceptible *S. aureus* (MSSA) and penicillin-susceptible *S. aureus* (PSSA) are typically treated with (flu)cloxacillin monotherapy. Methicillin-resistant *S. aureus* (MRSA) is typically treated with vancomycin monotherapy.

Alternative treatments, some specific to the infecting bacterium, are hypothesised to be either superior or, in some cases, non-inferior to standard practice. As summarised in Tong et al. [3], observational studies suggest that penicillin for PSSA [4], and cefazolin for MSSA [5, 6], may improve clinical outcomes compared to standard treatment. To treat MRSA, a randomised clinical trial found that combining vancomycin with an anti-staphylococcal penicillin did not improve survival but did decrease the occurrence of acute kidney injury [7]. The use of adjuvant therapies, such as clindamycin, is recommended in some guidelines for severe staphylococcal sepsis and may be effective against all *S. aureus* bloodstream infections but has not yet been shown to improve outcomes in randomised clinical trials [8]. There is also some evidence that an early switch from intravenous to oral antibiotics among patients with a good initial response to therapy, which is desirable from both clinical, logistical, and societal perspectives, may be possible without compromising survival [9, 10].

But trialling treatments for *S. aureus* bloodstream infection is difficult because the disease varies both geographically and in terms of antibiotic resistance, and because diagnostic and therapeutic approaches vary considerably [3]. Recruiting patients onto multiple fixed-size trials in sufficient numbers to make reliable conclusions is therefore exceedingly difficult. The *Staphylococcus aureus* Network Adaptive Platform (SNAP) trial overcomes these difficulties through its platform trial design.

Platform trials are becoming an increasingly used study design that can determine the efficacy of the multiple different interventions across a number of different intervention modalities, for multiple different patient populations, within a single study, and in parallel [11–16]. The platform trial design is therefore ideal for the study of multi-faceted therapeutic approaches for a multi-faceted disease such as *S. aureus* bloodstream infection.

Specifically, the SNAP trial aims to investigate the effect of different interventions nested within three different treatment modalities or ‘domains’ on a primary endpoint of 90-day all-cause mortality for patients with either PSSA, MSSA, or MRSA bloodstream infection. [17] The three treatment domains comprise a *backbone antibiotic* domain, comprising antibiotics specific to the infecting *S. aureus * bacterium, an *adjunctive antibiotic* domain, comprising antibiotics applicable to all infecting *S. aureus* bacteria, and an *early oral switch* domain, focusing on early switching of antibiotics from intravenous to oral delivery routes. Importantly, the SNAP trial will include both children and adults, with adult data used to support inference for the paediatric populations (and vice versa). This design feature can be seen as a response to the discussion provided by Murthy, Fontela, and Berry [18] that Bayesian modelling of adults and paediatrics is a potential solution to the issue of paediatric decision-making being largely based on ad hoc results that are extrapolated from adult populations.

The SNAP trial design has the following key statistical features:

- *Multifactorial design*. Many interventions are evaluated, both individually and in combination with one another.
- *Bayesian hierarchical inference*. Intervention effects are modelled by a hierarchical Bayesian probability model [19–21]. Inference is improved by leveraging information across different disease and/or population subgroups.
- *Scheduled and frequent Bayesian analyses and decision rules*. Trial data are analysed and operating decisions are made, at scheduled instances, as efficacy information becomes available instead of waiting for the trial to finish [22], without compromising the integrity of the trial design or the statistical inference [18, 21]. Based on the results of scheduled analyses and prespecified decision rules, if an intervention is shown to be clearly efficacious, randomisation is will be ceased for that domain and the results declared publicly. Inferior interventions will be dropped from the study, possibly to be replaced with other promising candidates. Similarly, to conserve trial resources, an intervention arm may be stopped for statistical *futility* if there is a low probability of demonstrating efficacy [23].
- *Response-adaptive randomisation*. In certain cases, based on the results of the scheduled analyses, future participants may have a higher probability of being randomised to interventions that appear to be more efficacious [24, 25].

This paper summarises the statistical principles of the SNAP trial design as described in the SNAP trial core protocol [3], the document that details the central aims of a trial along with core trial endpoints, decision rules, and trial governance structures; domain-specific appendices, appendices to the core protocol describing the protocol relating to a given domain; and statistical analysis appendix, a comprehensive appendix to the core protocol that specifies, in general terms, the randomisation strategies and statistical model/s that will be used to analyse the trial data (see https://www.snaptrial.com.au). Particular focus is given to the statistical methods that will be used to analyse the SNAP trial data along with the decision rules that will guide the trial adaptations.

This paper proceeds by describing, in general, the trial structure, statistical models, randomisation strategy, and trial decision making approach. We follow by describing the specifics of the trial in its initial configuration. A concluding discussion follows that places the SNAP design in the context of other platform trial designs.

## Trial structure

In broad terms, the SNAP trial model is designed around several ‘structural’ trial elements: silos, domains, subdomains, subgroups, covariates, regions and countries, and epochs. In a slight abuse of notation, for simplicity, we will often use *k* when referring to an integer-valued index. For example, *k* might index a domain or the levels of a covariate, depending on the context.

### Silo

A *silo* is a group of participants who are defined by the antibiotic susceptibility of their infecting isolate (e.g. MSSA, PSSA, and MRSA, see Table 1). Generally, a silo is denoted by *s*, which takes on an integer value if referring to a specific silo:

1 $$\begin{aligned} \{s \in S : S = 1, 2, 3, ..., n_S\}, \end{aligned}$$

where $n_S$ is the total number of silos. Silos are mutually exclusive and new silos are able to be accommodated as part of the trial design.

**Table 1:** Interventions and stopping rules

		**Domain (** $\boldsymbol{\mathcal{D}}$**)**		
		Backbone antibiotic (${\boldsymbol D}_{\mathbf1}$)	Adjunctive antibiotic (${\boldsymbol D}_{\mathbf2}$)	Early oral switch (${\boldsymbol D}_{\mathbf3}$)
**Strata (***S***)**	MSSA ($s=1$)	$$\begin{array}{c} D_{11}=\\ \left\{ \begin{array}{l} d_{11}:\text {(Flu)cloxacillin}* \\ d_{12}:\text {Cefazolin} \\ \end{array}\right. \\ {Non\text {-}inferiority} \end{array}$$	$$\begin{array}{c} D_{21}=\\ \left\{ \begin{array}{l} d_{21}:\text {No clindamycin}* \\ d_{22}:\text {Clindamycin} \\ \end{array}\right. \\ {Superiority} \end{array}$$	$$\begin{array}{c} D_{31}=\\ \left\{ \begin{array}{l} d_{31}:\text {Usual care}* \\ d_{32}:\text {Early oral switch} \\ \end{array}\right. \\ {Non\text {-}inferiority} \end{array}$$
	PSSA ($s=2$)	$$\begin{array}{c} D_{12} =\\ \left\{ \begin{array}{l} d_{11}:\text {(Flu)cloxacillin}* \\ d_{13}:\text {Penicillin} \\ \end{array}\right. \\ {Non\text {-}inferiority} \end{array}$$	$$\begin{array}{c} D_{22}=\\ \left\{ \begin{array}{l} d_{21}:\text {No clindamycin}* \\ d_{22}:\text {Clindamycin} \\ \end{array}\right. \\ {Superiority} \end{array}$$	$$\begin{array}{c} D_{32}=\\ \left\{ \begin{array}{l} d_{31}:\text {Usual care}* \\ d_{32}:\text {Early oral switch} \\ \end{array}\right. \\ {Non\text {-}inferiority} \end{array}$$
	MRSA ($s=3$)	$$\begin{array}{c} D_{13}=\\ \left\{ \begin{array}{l} d_{14}:\text {Vancomycin}* \\ d_{15}:\text {Vancomycin} + \text {cefazolin} \\ \end{array}\right. \\ {Superiority} \end{array}$$	$$\begin{array}{c} D_{23}=\\ \left\{ \begin{array}{l} d_{21}:\text {No clindamycin}* \\ d_{22}:\text {Clindamycin} \\ \end{array}\right. \\ {Superiority} \end{array}$$	$$\begin{array}{c} D_{33}=\\ \left\{ \begin{array}{l} d_{31}:\text {Usual care}* \\ d_{32}:\text {Early oral switch} \\ \end{array}\right. \\ Non\text {-}inferiority \end{array}$$

Stopping rules are shown in *italics*. If a non-inferiority rule is met, a decision to continue recruitment (to seek a superiority conclusion) may be made by the data safety and monitoring committee. Futility stopping rules also apply. Asterisks indicate reference interventions

*MRSA,* methicillin-resistant *S. aureus*; *MSSA*, methicillin-susceptible *S. aureus*; *PSSA*, penicillin-susceptible *S. aureus*

### Domains

A *domain* defines a set of mutually exclusive, competing interventions sharing a common clinical mode of action or clinical context of use (e.g. backbone antibiotics, adjunctive antibiotics, and early oral switch, see Table 1). The number of domains and the number and identity of individual interventions within each of these domains may vary across the life of the platform as inferior interventions are abandoned, superior interventions are identified, and/or new interventions of interest are included. Participants may be randomised to an intervention within each domain, but some participants may not be eligible to be randomised to all domains.

Domains are denoted by $D_k$, where *k* indexes a particular domain, and are contained with the set of all domains in the trial $\mathcal {D}$:

2 $$\begin{aligned} \{D_k \in \mathcal {D}: k = 1, 2, 3, ..., n_{\mathcal {D}}\}, \end{aligned}$$

where $n_\mathcal {D}$ is the total number of domains. Interventions within a particular domain are labelled using a lower-case *d* that is indexed by domain-specific subscript $\{j \in J_{D_k}: J_{D_k} = 1,2,3,..., n_{D_k}\}$, where $n_{D_k}$ is the total number of interventions in domain $D_k$. Under this nomenclature, $d_{kj}$ refers to intervention *j* within domain $D_k$.

### Subdomain

For participants within a specific silo, it is possible for the set of allocated interventions within each domain to be a subset of the full set of interventions available for that domain. We define the set of these silo-specific domain interventions by:

3 $$\begin{aligned} \{D_{ks} \subseteq D_k: k = 1, 2, 3, ..., n_{\mathcal {D}}; s = 1, 2, 3, ..., n_S\}. \end{aligned}$$

This silo-specific set of domain interventions leads us to the definition of a *subdomain*. For example, we might have $D_1 = \{d_{11}, d_{12}, d_{13}\}$ with $D_{11} = \{d_{11}, d_{12}\}$ and $D_{12} = \{d_{11}, d_{13}\}$ or indeed even $D_1 = D_{11} = D_{12}$. We define a subset $D^*_{ks}$ that omits the silo-specific domain reference intervention from $D_{ks}$ so that we can form the appropriate contrasts in the primary model.

### Subgroups

A *subgroup* refers to some mutually exclusive population characteristic, other than silo membership or Covariates, into which the platform participants can be partitioned and for which subgroup-specific estimates of treatment effect are of interest (e.g. adults and children). Domain efficacy can be assessed separately for these subgroups, possibly by silo, either by stratification or modelling. Details will be stated in the relevant domain-specific appendix. Subgroups of a particular characteristic are denoted by *u*, which takes an integer value if referring to a specific subgroup:

4 $$\begin{aligned} \{u \in U : U = 1, 2, 3, ..., n_U\}, \end{aligned}$$

where $n_U$ is the total number of subgroups. Note that we only consider a single characteristic in this trial design, age group (i.e. adult vs paediatric). Additional subgroups could be accommodated by expanding the definition in (4) such that $\{U_k \in \mathcal {U} : k = 1,2,3,..., n_{\mathcal {U}}\}$, where $\mathcal {U}$ denotes the set of all subgroups, $n_\mathcal {U}$ is the total number of subgroups and $U_k$ denotes the subgroup.

### Covariates

We define *covariates* as discrete baseline variables, other than silo membership or subgroup characteristics, which are likely to have some prognostic value. A covariate is denoted by $Z_k$, where *k* indexes a particular covariate, and is contained within the set $\mathcal {Z}$ of all possible covariates:

5 $$\begin{aligned} \{Z_k \in \mathcal {Z} : k = 1, 2, 3, ..., n_{\mathcal {Z}}\}. \end{aligned}$$

Values of a particular covariate are labelled by the lower-case letter associated with that covariate indexed by covariate specific subscript $j = \{1,2,..., J_{Z_k}\}$. For each covariate, we define a subset $Z^*_{k}$ that omits the reference value, typically the most frequently observed, from the subset.

### Regions and countries

A *region* is defined here as a broad geographic region, for example, Oceania:

6 $$\begin{aligned} \{r \in R: R = 1,2,3,...,n_R\}, \end{aligned}$$

where *r* denotes the region within the set of all $n_R$ regions *R*. We define a subset $R^*$ that omits the reference region from *R*.

We define *countries* as nested within regions, such that:

7 $$\begin{aligned} \{c_r \in C_r: C_r = 1, 2, 3, ..., n_{C_r}\}, \end{aligned}$$

where $c_r$ denotes a country belonging to the set of all $n_{C_r}$ countries $C_r$ within region *r*.

### Epochs

The concept of an *epoch* allows any advances in medical practice or changes in the virulence of *S. aureus* isolates with time to be accounted for across the lifetime of a multi-year trial. Not accounting for time in an adaptive trials where parameters are estimated from data with control cohorts that were randomised at different times can, in some circumstances, introduce biases and increase type I error or reduce power [26, 27]. An epoch is defined here as the calendar time corresponding to a 26-week period:

8 $$\begin{aligned} \{t \in T: T = 1,2,3,...,n_T\} \end{aligned}$$

where the number *t* corresponds to the natural temporal ordering of epochs, and $n_T$ represents most recent 26 week period for the purposes of analysis.

## Statistical modelling

The SNAP trial data will be analysed using Bayesian statistical methods, which combine probability distributions that summarise the state of knowledge independent of the observed data (a prior probability distribution) with the observed data model (through a likelihood function) to produce probability distributions that reflect an updated state of knowledge (a posterior probability distribution).

### Models

#### General linear function

The SNAP trial core protocol and its appendices specify multiple different endpoints for analysis, including binary, continuous, time-to-event, and ordinal endpoints. Here, we define the general linear function that will be used to model these endpoints using the appropriate linking functions. Note that we use the notation *s*(*i*), *u*(*i*) to denote the silo and subgroup to which participant *i* belongs, respectively. Similarly for participant *i*, $d_{kj(i)}$ indicates the treatment received, $D_k(i)$ indicates domain non-membership, $z_{kj(i)}$ indicates covariate level, *r*(*i*) and $c_r(i)$ indicate region and country location, and *t*(*i*) indicates epoch during which they were randomised. The general linear function is defined as follows:

9 $$\begin{aligned} f_i&= \alpha _{s(i), u(i)} + \sum \limits _{k_E \le n_\mathcal {D}} \beta _{s(i),u(i),d_{kj(i)}} + \sum \limits _{(k_E < l_E, l_E \le n_\mathcal {D})} \psi _{s(i),u(i),d_{kj(i)},d_{lj(i)}} \nonumber \\&\quad +\sum \limits _{k_I \le n_\mathcal {D}} \gamma _{D_k(i)} + \sum \limits _{k \le n_\mathcal {Z}}\theta _{Z_k, z_{kj(i)}} + \delta _{r(i)} + \omega _{c_r(i)} + \phi _{t(i)}. \end{aligned}$$

The parameters described in (9) are defined, for a general endpoint, as follows:

- $\alpha _{s,u}$—for participants in silo *s* and subgroup *u*, the value for eligible reference interventions, covariate level $z_k \notin Z^*_k$, region $r \notin R^*$, and current epoch.
- $\beta _{s,u,d_{kj}}$—for participants in silo *s* and subgroup *u*, the effect of intervention $d_{kj}\in D_{ks}^*$ compared to the reference intervention. Note that only parameters referencing eligible domains are included, where eligible domains for participant *i* are indexed by $k_E$.
- $\psi _{s,u,d_{kj},d_{kl}}$—for participants in silo *s* and subgroup *u*, the effect of the interaction between an intervention $d_{kj} \in D_{ks}^*$ with an intervention $d_{lj} \in D_{ls}^*$, where *k* is less than *l*. Note that only parameters referencing eligible domains are included, where eligible domains for participant *i* are indexed by $k_E$ (and likewise, $l_E$). Also note that the parameter is specific to two-way interactions only.
- $\gamma _{D_k}$—for all participants, the effect of ineligibility for domain $D_k$. Note that only parameters referencing ineligible domains are included, where ineligible domains for participant *i* are indexed by $k_I$.
- $\theta _{Z_k,z_{kj}}$—the effect of covariate factor $z_{kj} \in Z^*_{k}$, for all covariates $Z_k$.
- $\delta _r$—the effect of the region $r \notin R^*$ in which the participant is located.
- $\omega _{c_r}$—the effect of the country (that is nested within region) in which the participant *i* is located.
- $\phi _t$—for participants the current 26 week epoch, where epochs are contiguous, the effect of time *t*.

#### Primary endpoint model

The SNAP trial primary endpoint is binary, denoted $y \in \{0,1\}$, and is modelled using Bernoulli distribution with a logistic link function such that:

10 $$\begin{aligned} y_i&\sim {\textrm{Bernoulli}}(\pi _i)\nonumber \\ \pi _i&= {\textrm{logit}}^{-1}\left( f_i\right) , \end{aligned}$$

where $\pi$ is the probability of the event conditional on the terms described in (9).

We interpret the $\alpha _{s,u}$ in (10) as the log-odds of the 90-day all-cause mortality for eligible domain reference intervention in silo *s* and subgroup *u* for the reference covariates and the $\beta _{s,u,d_{kj}}$ as the log-odds ratio of the 90-day all-cause mortality, relative to the reference intervention, of domain interventions $d_{kj}$ in silo *s* and subgroup *u*.

### Prior distributions and model hierarchy

**Fig. 1:**
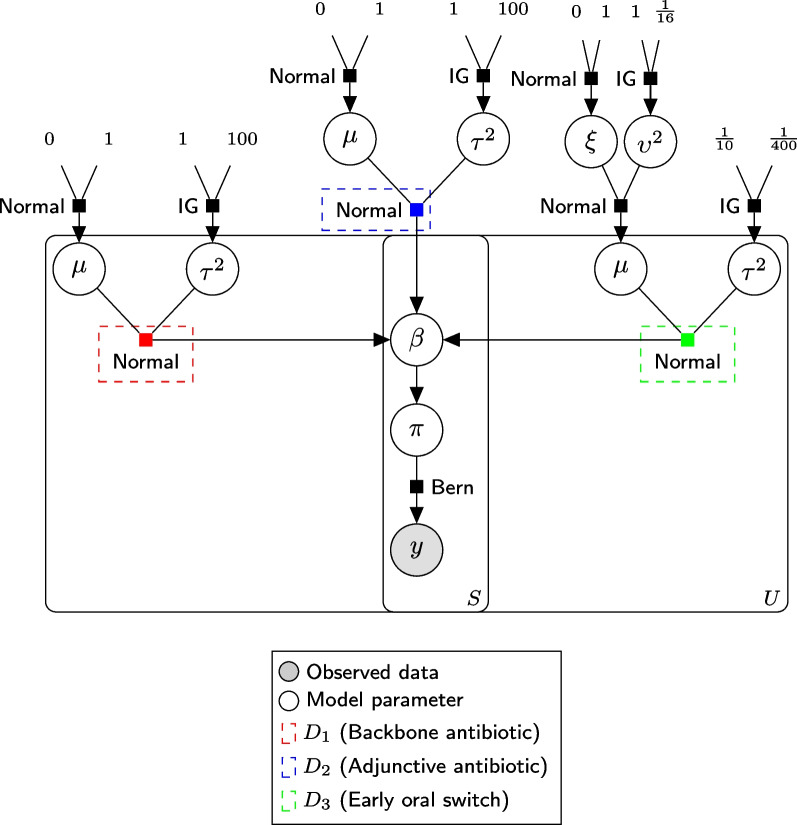
Graphical model of primary endpoint (simplified to intervention parameters only). IG, inverse-gamma

The following subsections outline the prior distribution structure for the parameters of the model using a Bernoulli distributed endpoint and logistic link function, including the primary model. The model for alternatively distributed endpoints will require an alternative prior structure, which will be described in detail in an openly available, domain-specific statistical analysis plan that will be published prior to the closure of a domain for terminal analysis. For clarity, a simplified graphical model is provided for the primary endpoint in Fig. 1. The following presentation is general; however, the fixed values chosen by investigators for the SNAP trial are described in later sections and in Table 2.

**Table 2:** Prior distributions and model hierarchy

Parameters	Normal$\boldsymbol(\boldsymbol a\boldsymbol,\boldsymbol b^{\mathbf2}\boldsymbol)$		Inv-Gamma(*p*, *q*)	
	*a*	*b*	*p*	*q*
Reference incidences (11)				
$\alpha _{s,u}$	-2	10	–	–
Effects of intervention				
*Silo-specific* (12)				
$\mu _{{s,d_{kj}}}$	0	1	–	–
$\tau ^2_{{s,d_{kj}}}$	–	–	1	1/16
*Subdomain-fixed* (13)				
$\mu _{{d_{kj}}}$	0	1	–	–
$\tau ^2_{{d_{kj}}}$	–	–	1	1/16
*Subdomain-exchangeable* (14)				
$\tau ^2_{u,d_{kj}}$	–	–	1/10	1/400
$\xi _{{d_{kj}}}$	0	1	–	–
$\upsilon ^2_{{d_{kj}}}$	–	–	1	1/16
Effects of domain eligibility (15)				
$\gamma _{D_k}$	0	1	–	–
Effects of two-way interactions (16)				
$\psi _{s,u,d_{kj}, d_{k'j}}$	0	1	–	–
Effects of regions and countries (17), (18)				
$\delta _r$	0	10	–	–
$\tau ^2_r$	–	–	1	1/16
Effects of covariates (19)				
$\theta _{Z_k,z_k}$	0	10	–	–
Effects of epoch (20)				
$\tau ^2_t$	–	–	1/4	1/10

In-text priors are cross-referenced by the first column parentheses

#### Reference log-odds

The log-odds for domain-specific interventions $d_{kj}$ are defined for each silo *s* and for each subgroup *u* and are assigned independent normal prior distributions as follows:

11 $$\begin{aligned} \alpha _{s,u} \sim \mathcal {N}(a, b^2), \end{aligned}$$

where *a* and *b* are fixed values set by the investigators to be weakly-informative for all silos and subgroups.

#### Effects of interventions

The log-odds *ratios* for domain-specific interventions $d_{kj}$ are similarly defined for each silo *s* and for each subgroup *u*; however, the hierarchical prior structure depends on the a priori assumptions of exchangeability of these parameters across silos. Irrespective of the different exchangeability assumptions, the prior structure is always hierarchical in that it allows information between subgroups (i.e. adult and paediatric groups) to be shared. The different prior structures are summarised as follows:

- Where a silo has a unique subdomain (i.e. it exists only for a single silo), the log-odds ratios where the intervention is silo-specific are modelled as normally distributed such that all subgroups have the same silo-specific mean and variance:
  12 $$\begin{aligned} \beta _{s,u,d_{kj}}&\sim \mathcal {N}({\mu_{s, d_{kj}}}, \tau ^2_{{s,d_{kj}}})\nonumber \\ \mu _{{s, d_{kj}}}&\sim \mathcal {N}(a,b^2)\nonumber \\ \tau ^2_{{s,d_{kj}}}&\sim \text {Inv--Gamma}(p,q), \end{aligned}$$
  where *a*, *b*, *p*, and *q* are fixed values set by the blinded investigators. We refer to this prior structure as ‘silo-specific’.
- Where two or more silos share a subdomain, and the log-odds ratios are a priori considered be common across silos (i.e. $\beta _{s,u,d_{kj}} = \beta _{u,d_{kj}}$ for all *s*), the silo- and subgroup-specific log-odds ratio for an intervention may be modelled as normally distributed with an intervention-specific mean and variance:
  13 $$\begin{aligned} \beta _{u,d_{kj}}&\sim \mathcal {N}(\mu _{{d_{kj}}}, \tau ^2_{{d_{kj}}})\nonumber \\ \mu _{{d_{kj}}}&\sim \mathcal {N}(a,b^2)\nonumber \\ \tau ^2_{{d_{kj}}}&\sim \text {Inv--Gamma}(p,q), \end{aligned}$$
  where *a*, *b*, *p*, and *q* are fixed values set by the blinded investigators. We refer to this prior structure as ‘subdomain-fixed’.
- Where two or more silos share a subdomain, and the log-odds ratios are a priori considered to be exchangeable, the log-odds ratios are modelled as normally distributed with a subgroup-specific mean and variance, with the subgroup-specific mean modelled as normally distributed with an intervention-specific mean and variance:
  14 $$\begin{aligned} \beta _{s,u,d_{kj}}&\sim \mathcal {N}(\mu _{{u, d_{kj}}}, \tau ^2_{{u,d_{kj}}})\nonumber \\ \mu _{{u, d_{kj}}}&\sim \mathcal {N}(\xi _{{d_{kj}}}, \upsilon ^2_{{d_{kj}}})\nonumber \\ \xi _{{d_{kj}}}&\sim \mathcal {N}(a,b^2)\nonumber \\ \tau ^2_{{u,d_{kj}}}&\sim \text {Inv--Gamma}(p,q)\nonumber \\ \upsilon ^2_{{d_{kj}}}&\sim \text {Inv--Gamma}(p*,q*), \end{aligned}$$
  where *a*, *b*, *p*, *q*, $p*$, and $q*$ are fixed values set by the investigators. This hierarchical prior structure ensures that the effect estimates for interventions in each silo will be ‘shrunk’ toward one another. Note that $p*$ and $q*$ are not necessarily equal to *p* and *q*, respectively. We refer to this prior structure as ‘subdomain-exchangeable’.

#### Effects of domain eligibility

The log-odds ratios for domain ineligibility are assigned independent normal prior distributions such that:

15 $$\begin{aligned} \gamma _{D_k} \sim \mathcal {N}(a,b^2), \end{aligned}$$

where *a* and *b* are fixed values set by the blinded investigators.

#### Effects of two-way interactions

The log-odds ratios for two-way interactions are assigned independent normal prior distributions such that:

16 $$\begin{aligned} \psi _{s,u,d_{kj},d_{k'j}} \sim \mathcal {N}(a,b^2), \end{aligned}$$

where *a* and *b* are fixed values set by the blinded investigators and *k* is less than $k'$. Where an interaction is considered a priori implausible, the interaction terms are set simply to zero.

#### Effects of regions and countries

The log-odds ratios for regions are assigned normal distributions such that:

17 $$\begin{aligned} \delta _r \sim \mathcal {N}(a,b^2), \end{aligned}$$

where *a* and *b* are fixed values set by the investigators. The log-odds ratio of country are nested within region hierarchically and are treated as exchangeable within region such that:

18 $$\begin{aligned} \omega _{c_r}&\sim \mathcal {N}(0, \tau _r^2)\nonumber \\ \tau _r^2&\sim \text {Inv--Gamma}(p,q) \end{aligned}$$

where *p*, and *q* are fixed values set by the blinded investigators.

#### Effects of covariates

The log-odds ratios for covariate factors are assigned independent normal prior distributions such that:

19 $$\begin{aligned} \theta _{Z_k,z_{kj}} \sim \mathcal {N}(a,b^2), \end{aligned}$$

where *a* and *b* are fixed values set by the blinded investigators.

#### Effects of epoch

Temporal trends will be accounted for by splitting the trial sample into separate cohorts defined by contiguous 26 week epochs and using first-order dynamic normal linear model within (9) and broadly suggested by [26, 27]:

20 $$\begin{aligned} \phi _{n_T}&=0\nonumber \\ \phi _{t-1}&\sim \mathcal {N}(\phi _t, \tau _t^2)\nonumber \\ \tau _t^2&\sim \text {Inv--Gamma}(p,q) \end{aligned}$$

where *p*, and *q* are fixed values set by the blinded investigators.

Furthermore, an additional sensitivity analysis may be performed to evaluate all outcomes of interest using cohorts that are restricted to participants who were randomised concurrently among the available interventions at the time and removing the $\phi_t$ parameter from the model.

### Exploratory analyses

Additional analyses are described in the SNAP statistical appendix and relevant domain-specific appendices that include parameters for the interactions between interventions and other covariates to enable the comparative effectiveness by covariates as a pre-planned exploratory analysis.

### Computational methods

The joint posterior probability distributions of the model parameters described in the preceding sections are analytically intractable, and therefore computational Bayesian methods will be used for the data analyses. We will use Markov-chain Monte Carlo (MCMC) methods implemented in stan, a probabilistic programming language [28], to numerically compute the joint posterior distributions of the parameters for each model based on the likelihood functions for the models and prior parameter distributions. Specifically, we will sample from the posterior distribution of each parameter of the model by using the Hamiltonian Monte Carlo algorithm implemented within stan, called from the R software environment [29]. For each parameter, we will run three or more MCMC chains in parallel for a ‘burn-in’ phase and sampling phase for as many iterations that are sufficient for the SNAP trial analytic team to be confident in the inference. Convergence of the MCMC chains will be assessed by the SNAP trial analytic team via the effective sample size, the scale reduction $\hat{R}$ , and graphical representations of the MCMC chains [30].

### Missing data

Death from *S. aureus* bloodstream infection predominantly occurs prior to hospital discharge, therefore, participants that are discharged alive from hospital but are lost to follow-up prior to day 90 (i.e. have missing outcome data) will be assumed to be alive at day 90, unless later information becomes available indicating a death in the 90-day period. A complete case strategy will be used for all other missing outcome data.

### Concurrently randomised cohorts

By design, the way that participants are assigned to treatments in adaptive randomised trials changes over time, which can lead to treatment-outcome confounding in certain cases. For when that confounder is based on calendar time, we have included epoch modelling as one countermeasure to confounding (see the ‘Epochs’ section). An additional potential for confounding may occur when a participant is unable to be randomised to a particular *intervention* within a subdomain. Reasons why might include contraindication or site unavailability for that particular intervention. Currently, the SNAP trial only randomises participants among two treatments per subdomain, and therefore the primary model is sufficient. In the event that the participant is to be randomised among more than two treatments, the SNAP study team may include additional model parameters to capture the ineligibility of participants to particular interventions.

As a further safeguard against treatment-outcome confounding arising from changing randomisation schemes, at the final analysis of a domain, we intend to perform and report sensitivity analyses of the primary model outcomes of each embedded fixed design corresponding to concurrently randomised cohorts, as recommended [31]. A concurrently randomised cohort is a subset of trial participants who had the same treatments available and all had the same chance of receiving those treatments (through randomisation) over the same time period.

## Randomisation

### General principles

Consented participants will be randomly allocated among interventions in subdomains for which they are eligible. Participants will be randomised to one intervention from each domain that their enrolling site is participating in, according to allocation probabilities detailed in each domain-specific appendix and stratified by subgroup. Although patients are randomised to receive an intervention drawn from each element of $D_{ks}$ at platform entry, since the eligibility for some silos and domains may not be known at that time, allocations in some domains may not be revealed until the participant fulfils the relevant silo and domain eligibility rules.

### Response-adaptive randomisation

Response-adaptive randomisation [24, 25] may be applied at the silo and subdomain level if a silo has a subdomain comprising at least three active intervention arms. Where response-adaptive randomisation is applied, then assignment probabilities for all interventions within the subdomain will initially be equal. Upon reaching the next scheduled analysis, data accumulated on the primary endpoint will subsequently guide allocation probabilities. For each subgroup, randomised assignment probabilities among the subdomain interventions will be permitted to vary proportional to the probability that each intervention is the most effective within that subdomain. Allocation probabilities will be based on the results of scheduled analyses.

Response-adaptive randomisation will be used to update randomisation probabilities as follows:

21 $$\begin{aligned} \rho _{s,u,d^*_{kj}} \propto \sqrt{\frac{P(\beta _{s,u,d^*_{kj}} = \min _j \beta _{s,u,d^*_{kj'}}) V(\beta _{s,u,d^*_{kj}})}{n_{s,u,d^*_{kj}}}}, \end{aligned}$$

where $j \ne j'$. Here, $\rho _{s,u,d^*_{kj}}$ is the updated randomisation probability for intervention $d^*_{kj}$ in subdomain $D_{ks}$ for subgroup *u*, $P(\beta _{s,u,d^*_{kj}} = \min _j \beta _{s,u,d^*_{kj'}})$ is the probability that intervention $d^*_{kj}$ is best of the non-reference interventions in domain $D_{ks}$ for subgroup *u*, $V(\beta _{s,u,d^*_{kj}})$ is the variance of the posterior distribution of $\beta _{s,u,d^*_{kj}}$, and $n_{s,u,d^*_{kj}}$ is the total number of participants with 90 days follow-up who have been allocated to an intervention $d^*_{kj}$ within a domain $D_{ks}$ for subpopulation *u*.

### Ineligible or unavailable domains

A participant may be ineligible for a specific domain because of contraindications (e.g. allergies, intolerances, adverse events) or non-contraindications (e.g. lack of access, clinician discretion, site opted out of domain). Domain ineligibilities will be incorporated by including the $\gamma _{D_k}$ terms of the statistical model in (9), which represent the incremental effect on the outcome of ineligiblity or unavailability of a domain for all participants. If a participant is ineligible for all interventions within a domain and/or an entire domain is unavailable at the site at the time of randomisation, then the participant will also be deemed ineligible for that domain.

If response-adaptive randomisation is used and a participant is ineligible for one or more interventions within a domain, assignment probabilities will be re-normalised across the remaining eligible interventions, as long as there are a minimum of two eligible interventions within that domain that are available to the participant. If an intervention within a domain is unavailable at the time of randomisation for site-specific reasons (e.g. temporary lack of access to the intervention drug), then assignment probabilities will be re-normalised across the remaining available interventions, as long as there are a minimum of two eligible interventions within that domain that are available to the participant. Data on both the primary and secondary endpoints in participants flagged as ineligible (refused) for some or all interventions will still be captured and available for analysis.

## Scheduled updates and decision rules

The SNAP trial is designed to be perpetual and answer both clinician and consumer priority-driven research questions for *S. aureus* bloodstream infection. At scheduled intervals, the available data will be used to update posterior distributions that, in turn, will be used to inform operational decision rules based on prespecified stopping rules.

### Data source

Allocation to randomised interventions remains concealed until the time it is revealed to site investigators. The assessment of primary and secondary outcomes by site investigators will not be blinded to knowledge of allocated intervention. All trial and site investigators will remain blinded to aggregated trial outcomes. Only the SNAP analytic team and data safety and monitoring committee members will have access to unblinded aggregated trial outcomes. Scheduled updates will be performed using all available data from eligible SNAP trial participants. Unblinded data will be extracted from the SNAP trial database immediately prior to each update and provided to the SNAP trial analytic team. The SNAP analytic team and data safety and monitoring committee, unblinded to treatment allocation, are independent of all other trial committees to ensure independence.

### Posterior summaries

The primary endpoint is all-cause mortality at 90 days after platform entry. The primary contrast of interest (i.e. the *estimand*) is the effect, within a given domain, of the intervention compared to the reference intervention on the probability of the primary endpoint, in randomised participants in the adult population. This effect will be summarised by the log-odds ratio of the primary endpoint computed from (10). Note that an odds ratio greater than one indicates an increase in mortality.

The full list of endpoints, population summaries, and populations within the estimand framework can be found in the core protocol, domain-specific appendices, and the statistical appendix (see https://www.snaptrial.com.au).

The posterior distributions of each parameter of interest will be summarised using medians and 95% credible intervals. A 95% credible interval is conceptually similar to a frequentist 95% confidence interval although is interpreted as the interval within which the parameter of interest will fall with 95% probability. We use equal-tailed credible intervals which take the 2.5th and 97.5th percentiles as their respective lower and upper bounds.

### Decision rules

The posterior distribution of the odds ratio for the primary endpoint forms the basis of the decision rules for concluding *superiority* or *non-inferiority* for an intervention or statistical *futility* of the efficacy objectives as described in the following subsections. Note that the thresholds used to define these decision rules were determined via extensive computer simulations, which are outlined in the ‘Trial operating characteristics’ section.

#### Superiority

Clinically, the superiority of any intervention $d_{kj}$ versus the reference intervention, unless specified otherwise, is defined for the adult population as an odds ratio of less than one for the primary endpoint. A stopping decision for superiority will be made if, at a scheduled update, the posterior probability of superiority in the relevant silo (or domain), for the adult subgroup, is greater than $q_E$:

22 $$\begin{aligned} \text {Pr}\left[ \exp (\beta _{s,u=1,d_{kj}}) < 1\right] > q_E. \end{aligned}$$

Similarly, the stopping decision for futility of the superiority objective will be made if, at a scheduled analysis, the posterior probability of superiority, for the adult subgroup, is less than $q_{E_F}$:

23 $$\begin{aligned} \text {Pr}\left[ \exp (\beta _{s,u=1,d_{kj}})< 1/1.2\right] < q_{E_F}. \end{aligned}$$

#### Non-inferiority

Clinically, the non-inferiority of any intervention $d_{kj}$ versus the reference intervention is defined for the adult population as an odds ratio of less than 1.2 for the primary endpoint. A stopping decision for non-inferiority will be met if, at a scheduled update, the posterior probability of non-inferiority in the relevant silo (or domain), for the adult subgroup, is greater than $q_{NI}$:

24 $$\begin{aligned} \text {Pr}\left[ \exp (\beta _{s,u=1,d_{kj}}) < 1.2\right] > q_{NI}. \end{aligned}$$

Similarly, the stopping decision for futility of the non-inferiority objective will be made if, at a scheduled analysis, the posterior probability of non-inferiority, for the adult subgroup, is less than $q_{NI_F}$:

25 $$\begin{aligned} \text {Pr}\left[ \exp (\beta _{s,u=1,d_{kj}})< 1.2\right] < q_{NI_F}. \end{aligned}$$

### Frequency of scheduled updates

Scheduled updates will be made each time that an additional 500 randomised participants, including both adults and children, have completed 90 days of follow-up from platform entry. Extensive simulations have led to this schedule that, along with prespecified decision rules, maintains a low probability of declaring a false-positive result (see the ‘Trial operating characteristics’ section) and ensures that enough data is collected to assess secondary safety outcomes that may have lower frequencies.

### Introducing new interventions

If a new intervention is introduced to an active subdomain (a subdomain with at least two interventions), then randomisation will be fixed for the new intervention in order to guarantee a sufficient allocation to the new intervention. If upon introducing the new intervention to a subdomain indexed by *ks* there are $n_{ks}$ interventions, then a restricted allocation of $1/n_{ks}$ will be used to allocate participants to the new intervention. The remaining $1-(1/n_{ks})$ probability will be allocated to the other interventions either equally or with a probability determined by response-adaptive randomisation and, at the subsequent scheduled update, either equal allocation between all interventions will continue or the restriction will be removed and response-adaptive randomisation will be applied to all interventions, depending on what is specified in the relevant domain-specific appendix.

### Trial conclusions and reporting

At each scheduled analysis, the SNAP trial analytic team will prepare an unblinded report for the data safety and monitoring committee. If stopping rules are satisfied, the data safety and monitoring committee will make recommendations to the global trial steering committee on the public disclosure of results and/or the removal of interventions within particular subdomains.

Note that if a stopping rule for superiority is met for a given subdomain, then the intention is to cease randomisation to all relevant interventions. All future trial participants may be deterministically allocated to the superior intervention, or a different intervention determined by the treating clinician, until a new intervention is added to the subdomain. If the treating clinician determines the intervention, then the choice will be recorded in the trial database. If non-inferiority is demonstrated at a scheduled analysis, then recruitment into that subdomain may continue in order to seek a conclusion of superiority, based on prespecified rules in the relevant domain-specific appendix, and the recommendation from the data safety and monitoring committee and global trial steering committee. Further safety outcomes are reviewed by the data safety and monitoring committee at each scheduled analysis who, within their charter, may request a continuation of enrolment, even if a non-inferiority or superiority decision criteria have been met, in order to collect more information on the safety of intervention(s).

### Changes to prespecified analyses

The unblinded SNAP trial analytic team will monitor the primary and secondary model behaviour, including numerical stability and scientific appropriateness. Alternative models may be needed if there are unforeseen numerical, data, or modelling problems. Any alternatives will be implemented by the SNAP trial analytic team and communicated to the data safety and monitoring committee and may be reported to the global trial steering committee providing that there is no risk to trial integrity.

## Initial implementation

### Trial structure

**Fig. 2:**
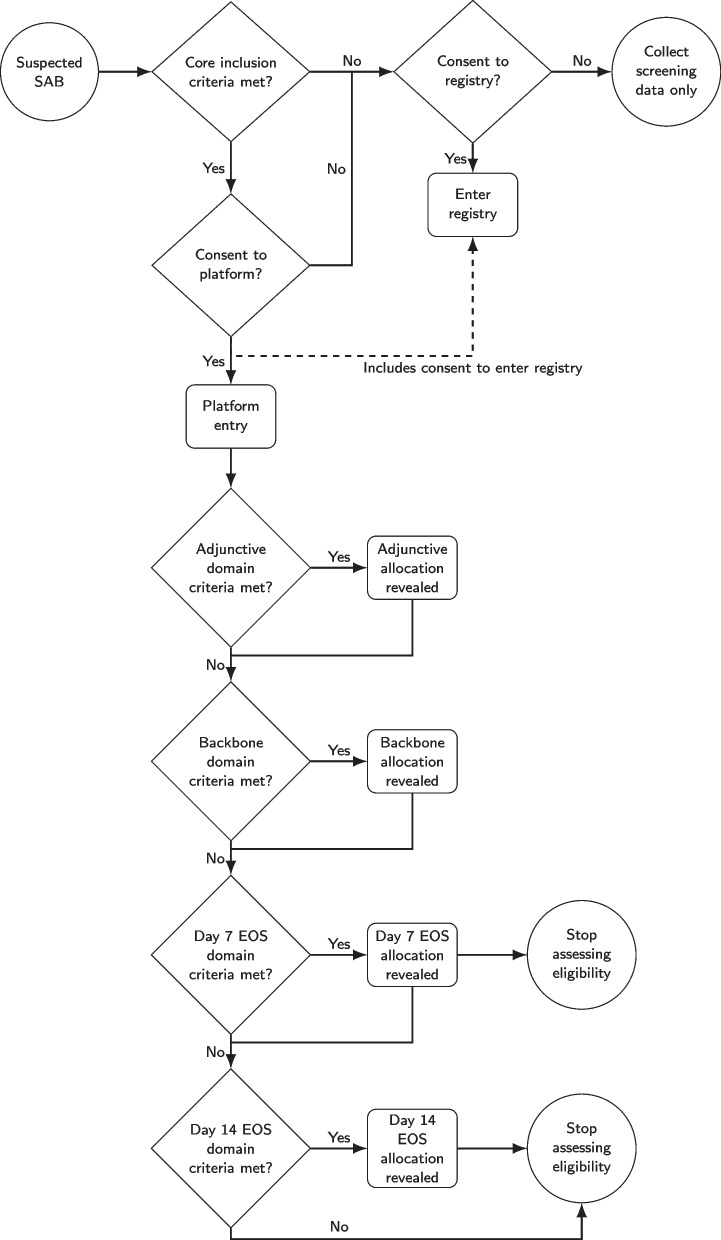
Participant flow, randomisation, and reveal sequencing. SAB, *Staphylococcus aureus bloodstream infection/bacteremia*; EOS, early oral switch

The initial SNAP trial silo, domain, and subdomain structure at commencement is summarised in Table 1. The participant flow, randomisation, and reveal sequencing are summarised in Fig. 2. Key features are as follows:

- The primary endpoint is all-cause mortality at 90 days after platform entry.
- Each subdomain initially consists of two interventions only.
- The subdomains of each silo within the backbone antibiotic domain are unique.
- For the adjunctive and early oral switch domains, subdomains are the same across silos.
- Only participants who have clinically stable disease and the ability to absorb or adhere to oral regimens, at day 7 for uncomplicated disease or day 14 for complicated disease will be eligible for the early oral switch domain.

The current design of the SNAP trial is optimised for comparative effectiveness. Future domains may allow the introduction of new interventions for registrational purposes. In such circumstances, the SNAP Global Trial Steering Committee will enter into discussions with the new product sponsors and the regulators regarding the details of the relevant domain-specific appendices, including specific concurrent control and safety reporting requirements.

### Primary model

The model of the primary endpoint is a special case of (10), with the linear component specified as follows:

26 $$\begin{aligned} f_i&= \alpha _{s(i), u(i)} + \sum _{k_E \le 3} \beta _{s(i),u(i),d_{1j(i)}} + \psi _{s(i) = 3,u(i),d_{1j(i)},d_{2j(i)}}\nonumber \\&\quad + \sum _{k_I \le 3} \gamma _{D_k(i)} + \theta _{Z_1, z_{kj(i)}} + \delta _{r(i)} + \omega _{c_r(i)} + \phi _{t(i)}. \end{aligned}$$

Note that the parameters in (26) are interpreted as described for (10). Silos, domains, and interventions are as described in Table 1. At platform commencement, only a single dichotomous subgrouping is specified for age such that $U=\{1,2\} \rightarrow \{\mathrm{`}\text{adult'}, \mathrm{`}\text{child'}\}$. Additionally, clinical plausibility leads us to consider only interactions between interventions in the MRSA silo in the backbone antibiotic and adjunctive antibiotic domains. Furthermore, a single covariate will be included in the primary model, age group, such that $Z_1 = \{1, ..., 9\} \rightarrow$ {‘30 days or less’, ‘31–365 days’, ‘1–4 years’, ‘5–11 years’, ‘12–17 years’, ‘18–39 years’, ‘40–59 years’, ‘60–79 years’, ‘80 years and over’}. Regions correspond broadly to established geopolitical regions such that $R = \{1, ...,8\} \rightarrow$ {‘Africa and the Middle East (excluding Israel)’, ‘East Asia’, ‘Europe (including Israel)’, ‘Oceania’, ‘North America’, ‘South and Central America’, ‘South-east Asia’, ‘Subcontinental Asia’}.

### Prior distributions and model hierarchy

The model hierarchy and prior structure is specified as detailed in the ‘Prior distributions and model hierarchy’ section. The specific choices for each prior parameter are provided in Table 2. In general, the priors are minimally informative.

### Decision rule thresholds

The decision rule thresholds are set at 0.99 for each of the superiority, $q_E$ (22), and non-inferiority , $q_{NI}$ (24), decision rules, and at 0.01 for each of the futility of superiority objective, $q_{E_F}$ (23), and futility of non-inferiority objective, $q_{NI_F}$ (25) decision rules.

### Trial operating characteristics

It is often possible to analytically derive frequentist trial operating characteristics such as power and type I error for simple trial designs using well known-formulae. For complicated trials such as the SNAP trial, no analytical formulae exist and therefore computer simulations are used to estimate the trial operating characteristics.

Extensive simulations were performed to evaluate the effect of different trial designs, such as the number and timing of scheduled analyses and decision rule thresholds, on the frequentist operating characteristics. The simulation process was iterative, involving close collaboration between the statistical and clinical team in which the trial design was refined over time by the SNAP Statistical Subcommittee. Table 3 summarises the proportion of 1000 simulated trials that satisfied the stated decision rules in each silo and domain for the decision rule thresholds stated in the previous subsection. In brief, the simulation assumptions were:

- A maximum sample size of 7000 (comprising 6000 adults and 1000 children)
- Proportion of patients in silos: MSSA (64%), PSSA (16%), and MRSA (20%)
- Reference mortality rates for adults and children in each silo, respectively, for participants who were not eligible for early oral switch: MSSA (15%, 2.2%), PSSA (15%, 2.2%), MRSA (20%, 3.5%)
- Proportion of participants eligible for early oral switch domain, for adults and children (respectively), at day 7 (10%, 45%) and day 14 (60%, 30%). Note that eligibility for early oral switch modified the reference mortality rates (as detailed in Additional file 1)
- Scenario 1 refers to the ‘null’ scenario in which the odds ratios were set equal to 1 where superiority was the main objective and 1.2 where non-inferiority was the main objective
- Scenario 2 refers to the ‘moderate’ treatment effect scenario where the odds ratio was set to 0.75 for all scenarios
- For the backbone antibiotic domain, decisions are based upon the ‘silo-specific’ effects of interventions (see (12)). For the adjunctive antibiotic domain, decisions are based upon the ‘subdomain-fixed’ effects of interventions (see (13)). For the early oral switch domain, decisions are based upon the ‘subdomain-exchangeable’ effects of interventions (see (14))

The proportions under scenario 1 correspond to piecewise frequentist type I errors, and we can see that for all silos and domains, the type I error is less than or equal to 7%. The proportions under scenario 2 correspond to the frequentist power of the trial. We can see that under a moderate treatment effect power is close to 80% or higher for non-inferiority rules except for the PSSA silo, the smallest silo, in the backbone antibiotic domain. Power for the MRSA silo in the backbone antibiotic domain is a relatively low 46% but not unexpected for the size of the silo. Power for superiority in the adjunctive antibiotic domain is 93%, noting that the decision rule for the domain is domain-specific, rather than silo-specific.

**Table 3:** Proportion of simulated trials declaring non-inferiority and superiority

Scenario	Strata	Non-inferiority			Superiority		
		Backbone	Adjunctive	EOS	Backbone	Adjunctive	EOS
1	MSSA	0.02	–	0.07	0.00	0.07	–
	PSSA	0.06	–	0.03	0.01	0.07	–
	MRSA	–	–	0.04	0.01	0.07	–
2	MSSA	0.99	–	0.95	0.77	0.93	–
	PSSA	0.61	–	0.78	0.29	0.93	–
	MRSA	–	–	0.84	0.46	0.93	–

Scenario 1 corresponds to an ‘all null’ scenario (odds ratio for non-inferiority: 1.2; odds ratio for superiority: 1.0); scenario 2 corresponds to ‘moderate effect’ scenario (odds ratio: 0.75 for all hypotheses)

*EOS*, early oral switch; *MSSA*, methicillin-susceptible *S. aureus*; *PSSA*, penicillin-susceptible *S. aureus.*; *MRSA*, methicillin-resistant *S. aureus*

The report detailing the full set of simulated trial operating characteristics, under a range of plausible scenarios, is available as an online supplement.

### Current state

The SNAP trial opened to recruitment on 16 February 2022. As of the date of the first scheduled analysis (31 May 2023), the trial has initiated all domains and treatments as described in Table 1 and enrolled 844 participants, over 59 sites, within 5 countries (Australia, Canada, Israel, New Zealand, and Singapore).

## Concluding remarks

We have provided a detailed description of the statistical principles of the SNAP trial. Rather than provide a comprehensive statistical analysis plan, our intention was to describe the trial design and structure using relatively formal language so that the different components of the trial could be referred to unambiguously. Our hope is that by formalising the platform trial structure we can encourage others to consider the value of platform trial designs in their research. To aid understanding, we have described the initial implementation of the SNAP trial design.

The SNAP trial is similar to other international platform trials currently underway such as the Australasian COVID-19 Trial (ASCOT) [32] and the Randomized, Embedded, Multifactorial Adaptive Platform trial for Community-Acquired Pneumonia (REMAP-CAP) [33]. A key differentiating feature is that the inferential model, and therefore clinical value of the SNAP trial, is strengthened by including both adult and paediatric populations. The approach of the SNAP trial design to modelling paediatric and adult data with common priors could conceivably be applied in other contexts where there are underrepresented population subgroups with substantially different phenotypes, for example in geriatric populations, in people who inject drugs, or biological sex.

Platform trials present unique and complex statistical challenges that cannot be addressed by a traditional statistical analysis plan. A flexible statistical model that can account for changes to the number of trial interventions, domains, and subgroups, and allow intervention comparisons that are balanced over eligibility criteria, covariates, and time, needs to be defined a priori. Such complexities are best addressed in a separate protocol statistical appendix that includes a summary of the simulations used to design the trial along with other statistical documentation including domain-specific statistical analysis plans that are implemented upon reaching a domain-specific conclusions. The protocol statistical appendix does not take the place of the statistical analysis plans that are required to report the results upon reaching a domain conclusion. Instead, the statistical appendix provide the template to implement the statistical model and decision rules at each scheduled analysis.

A consensus-based guideline/checklist is not yet available for this essential protocol content in platform trials. We believe that this publication serves as a first step toward a consensus on statistical documentation requirements for platform trials.

### Supplementary information

**Additional file 1.** SNAP Simulation Report.

## Data Availability

Not applicable.

## Abbreviations

COVID-19: Coronavirus disease of 2019
EOS: Early oral switch
MCMC: Markov-chain Monte Carlo
MRSA: Methicillin-resistant *S. aureus*
MSSA: Methicillin-susceptible *S. aureus*
PSSA: Penicillin-susceptive *S. aureus*
SNAP: *Staphylococcus aureus* Network Adaptive Platform trial
