# Supplementary material for: A blueprint for a multi-disease, multi-domain Bayesian adaptive platform trial incorporating adult and paediatric subgroups: the Staphylococcus aureus Network Adaptive Platform trial
Source: Trials. 2023 Dec 6;24:795. doi: 10.1186/s13063-023-07718-x (PMC10699085; doi:10.1186/s13063-023-07718-x)
Supplement: Supplementary file 1 — Additional file 1. SNAP Simulation Report. [file 13063_2023_7718_MOESM1_ESM.pdf]

SNAP Platform Trial

Simulation Report (Version 1)

February 14, 2023

Contents

1 Introduction **2**
- 1.1 Primary Endpoint . . . 2
- 1.2 Silos . . . 2
- 1.3 Domains . . . 2
- 1.4 Interventions . . . 3
- 1.5 Age Subgroups . . . 3

2 Statistical Modeling **3**
- 2.1 Model Priors . . . 4
  - 2.1.1 Baseline Mortality Rate . . . 4
  - 2.1.2 Intervention Effects . . . 4
  - 2.1.3 Age Group Effects . . . 5
  - 2.1.4 Reveal Effects . . . 5

3 Adaptive Design **6**
- 3.1 Interim Timing . . . 6
- 3.2 Statistical Triggers . . . 6

4 Example Trials **7**
- 4.1 Example Trial 1 . . . 8
- 4.2 Example Trial 2 . . . 16

5 Simulation Scenarios **23**
- 5.1 Baseline Patient Characteristics . . . 23
- 5.2 Accrual Rate . . . 23
- 5.3 Intervention Assignments . . . 23
- 5.4 Intervention Reveal . . . 23
- 5.5 Mortality Rate Scenarios . . . 25
  - 5.5.1 Reference Mortality Rate Scenarios . . . 25
  - 5.5.2 Treatment Benefit Scenarios . . . 26
  - 5.5.3 Simulated outcomes following a platform conclusion . . . 27

6 Operating Characteristics (Adult Age Group) **28**
- 6.1 All Equal (OR = 1.0) . . . 28
- 6.2 All Null . . . 29
- 6.3 All Effective (OR = 0.80) . . . 30
- 6.4 All Effective (OR = 0.75) . . . 31
- 6.5 All Effective (OR = 0.55) . . . 32
- 6.6 Harm Domain D_3 . . . 33
- 6.7 Mixed Domain D_3 . . . 34

7 Operating Characteristics (Both Age Groups) 36
7.1 All Equal (OR = 1.0) . . . 37
7.2 All Null . . . 37
7.3 All Effective (OR = 0.80) . . . 38
7.4 All Effective (OR = 0.55) . . . 38

8 Alternative Design Simulations 38

1 Introduction

SNAP is an adaptive platform trial evaluating a range of interventions to reduce mortality for patients with Staphylococcus *aureus* bacteraemia (SAB). The trial is built with the possibility of investigating multiple treatments within multiple domains.

An extensive set of simulations was performed during the process of constructing this adaptive platform trial. The selection of the model priors, adaptation timing, and decision thresholds were selected through simulations. In this report, we present the simulation results for the final set of design choices.

This simulation report is intended describe the operating characteristics of this trial design as estimated via trial simulation. It is not intended to substitute for a complete and final Statistical Analysis Plan. Details of the adaptive design and statistical model are found in the protocol and appendices (specifically the statistical appendix).

1.1 Primary Endpoint

The primary endpoint is 90-day mortality. The 90-day period is measured from the time of randomization. We label the outcome for a patient as Y, where $Y = 1$ is defined as an event (death within 90 days) and $Y = 0$ is a patient success.

1.2 Silos

The trial has three "silos" that differentiate groups of patients with different types of bacterium. Generally a silo is denoted by s, which takes on an integer value if referring to a specific silo:

$$s\epsilon S : S = 1, 2, 3, \dots n_S,$$

where n_S is the number of silos. Silos are mutually exclusive. At trial initiation, there are three silos:

- **PSSA** ($s = 1$): penicillin-susceptible S. aureus
- **MSSA** ($s = 2$): methicillin-susceptible S. aureus
- **MRSA** ($s = 3$): methicillin-resistant S. aureus

Adaptive decision rules will typically be applied separately within each silo, unless otherwise noted.

1.3 Domains

A domain is defined as a group of mutually exclusive, competing interventions with comparable modes of action or context of clinical care. Each domain contains a set of interventions among which a patient will be randomized. The trial is designed to be flexible and allow a fluctuating number of domains and interventions as the trial progresses. Domains are denoted by D_k where k indexes a particular domain.

In the simulations, we focus on the expected domains at the time of trial initiation:

- **Domain D_1:** Backbone antibiotic
- **Domain D_2:** Adjunctive antibiotic
- **Domain D_3:** Early oral stepdown (EOS)

1.4 Interventions

Each patient will be assigned to a single intervention within each domain. Interventions within a particular domain are labelled using a lower-case d that is indexed by domain-specific subscript. For the backbone antibiotic domain (Domain D_1), the set of available interventions depends on a patient's silo membership. Table 1 shows the set of available interventions within each domain and silo at trial initiation. Additional interventions and hypotheses may be added as the trial progresses.

Table 1: Interventions for each domain and silo

Silo	Intervention 1 (reference)	Intervention 2
**Domain D_1**		
PSSA	Flucloxacillin	Penicillin
MSSA	Flucloxacillin	Cefazolin
MRSA	Vancomycin	Vancomycin + Cefazolin
**Domain D_2**		
PSSA/MSSA/MRSA	No Clindamycin	Clindamycin
**Domain D_3**		
PSSA/MSSA/MRSA	Continued IV	Early oral switch

1.5 Age Subgroups

The trial will classify each participant into an age subgroup: adult or pediatric. A key feature of the statistical model is that the treatment effects for the two age subgroups are estimated with a hierarchical model that borrows information between subgroups.

2 Statistical Modeling

The adaptive aspects of the design are driven by a statistical model. Details of the statistical model are found in the protocol and appendices (specifically the statistical appendix). The model is written to be generalizable because the set of domains and interventions can evolve over time. For this report, the statistical model below conforms to the general structure but is written to reflect the concrete set of circumstances implemented in the simulations.

There are several instances in which the model and/or assumptions used for simulations are simplified from the expected implementation of the actual trial:

1. In the simulations, only Domain D_3 (early oral stepdown) allowed the possibility for a randomized assignment to never be revealed. Domains D_1 and D_2 assumed that every patient would have a randomized revealed assignment. The possibility of a patient and/or entire site declining participation in a domain was not simulated. Thus, in the statistical model used in the simulations, only Domain D_3 has an adjustment for whether the patient had a randomized, revealed assignment.
2. Actual patient ages were not simulated. In the actual trial, the model will adjust for finer grain age categories, but for the simulations, the model only broadly adjusts for adult versus child.

3. Interactions between interventions were neither modeled nor simulated.

The statistical model is structured so that an odds-ratio less than one corresponds to an improvement in mortality.

Let π_i be the 90-day mortality probability for patient i, which depends on:

- the patient's age group, $u = 1$ for adult, $u = 2$ for child;
- the patient's silo, s, which is either 1, 2, or 3 (for PSSA, MSSA, or MRSA respectively);
- whether the randomized assignment in Domain D_3 (early oral stepdown) was revealed; and
- the patient's randomized, revealed assignment in each domain.

We model the mortality probability as:

$$\log\left(\frac{\pi_i}{1-\pi_i}\right) = \alpha_{s(i),u(i)} + \sum_{\{D^*_{ks}\}} \beta_{s(i),u(i),d_{kj(i)}} + \gamma_{s(i),3}$$

The $\alpha_{s,u}$ parameter is the baseline log odds of death within 90 days for age group u in silo s randomized to the reference intervention in Domains D_1 and D_2 and with no revealed assignment in Domain D_3. It is expected that the mortality rate will vary across silos. For the simulations, we parameterize the baseline log odds for children as $\alpha_{s,2} = \alpha_{s,1} + \lambda$. Thus λ is the log-odds ratio on mortality for children relative to adults.

The $\beta_{s,u,d_{kj}}$ parameters are the global treatment effect parameters (log odds-ratios) for the investigational intervention j, relative to the reference intervention, for domain D_k within each age group u and silo s. The notation D^*_{ks} is the subset of interventions that omits the silo-specific domain control from domain D_{ks}. In Domain D_2 (adjunctive antibiotic), the treatment effect is a common estimate for all silos.

The $\gamma_{s,3}$ parameter captures the log odds-ratio for having a randomized, revealed assignment in Domain D_3 (early oral stepdown) relative to no revealed assignment. This effect may vary across silos.

2.1 Model Priors

In this section we present the prior distributions used for each of the parameters in the simulations.

2.1.1 Baseline Mortality Rate

The prior distribution for the log-odds of death for adults in each silo is

$$\alpha_{s,u=1} \sim N(-2, 10^2), \qquad s = 1, 2, 3.$$

2.1.2 Intervention Effects

Each $\beta_{s,u,d_{kj}}$ parameter is the relative effect of the investigational intervention compared to the reference intervention in the domain. All statistical triggers for the adaptive design are based on the posterior distributions of the odds-ratios, defined as OR $= \exp(\beta)$. The treatment effect may vary by silo s and by age group u. There is a hierarchical model that borrows information between adults and children so that the estimates of the treatment effect parameters are shrunk towards each other, with the degree of shrinkage depending on the observed similarity in the data.

In Domain D_1 (backbone antibiotic), the log odds-ratio within each age group is modeled separately per each silo:

$$\beta_{s,u,d_{1j}} \sim N(\mu_{s,D_1}, \tau^2_{s,D_1}), \qquad u = 1, 2; \qquad s = 1, 2, 3$$

with hyperpriors for the hierarchical borrowing between age groups:

$$\mu_{s,D_1} \sim N(0,1)$$

and

$$\tau^2_{s,D_1} \sim IG(1, 0.0625).$$

In Domain D_2 (adjunctive antibiotic), the treatment effect within each age group is a common estimate for all silos:

$$\beta_{u,D_2} \sim N(\mu_{D_2}, \tau^2_{D_2}), \qquad u = 1, 2$$

with hyperpriors for the hierarchical borrowing between age groups:

$$\mu_{D_2} \sim N(0,1)$$

and

$$\tau^2_{D_2} \sim IG(1, 0.0625).$$

In Domain D_3 (early oral stepdown), the treatment effect within each age group is estimated from a hierarchical model across silos and age groups:

$$\beta_{s,u,D_3} \sim N(\mu_{u,D_3}, \tau^2_{u,D_3}), \qquad u = 1, 2; \qquad s = 1, 2, 3$$

with a hyperprior on the variance that borrows across silos (within age group):

$$\tau^2_{u,D_3} \sim IG(0.25, 0.0025).$$

The mean for each age group is modeled with a hierarchical prior:

$$\mu_{u,D_3} \sim N(\xi_{D_3}, \tau^2_{D_3})$$

with

$$\xi_{D_3} \sim N(0,1)$$

and

$$\tau^2_{D_3} \sim IG(1, 0.0625)$$

The τ^2_{u,D_3} parameter is a variance parameter that controls the amount of shrinkage of the log odds-ratio estimates across silos within each age group for Domain D_3. The τ^2_{s,D_1}, $\tau^2_{D_2}$ and $\tau^2_{D_3}$ parameters are variance parameters that control the amount of shrinkage of the log odds-ratio estimates between adults and children. In Domain D_1 (backbone antibiotic), this borrowing happens within each silo separately. In Domain D_2 (adjunctive antibiotic), there is a single treatment effect estimate pooled across silos within each age group, and the model shrinks these estimates between adults and children. In Domain D_3 (early oral stepdown), there is a two-level hierarchical model that shrinks the treatment effect estimates for the silos in each age group, and also shrinks the estimates between adults and children.

2.1.3 Age Group Effects

The baseline mortality rate in children is expected to be much lower for children than for adults ($15 - 20\%$ for adults versus $2 - 3\%$ for children). The prior distribution is modestly informative with a mean expectation reflecting a substantial downward shift in the mortality rate for children versus adults:

$$\lambda \sim N(-1.5, 2^2).$$

2.1.4 Reveal Effects

The effect of having a revealed assignment in Domain D_3 (early oral stepdown) is modeled separately per silo with a weak prior:

$$\gamma_s \sim N(0,1), \qquad s = 1, 2, 3.$$

3 Adaptive Design

3.1 Interim Timing

Interim analyses occur after every 500 patients (total including both adults and children) have a known outcome for the primary 90-day mortality endpoint. In the simulations, interims continue on this schedule until a maximum sample size of 7000 patients is reached.

3.2 Statistical Triggers

At each interim, all available primary endpoint outcomes will be analyzed using the statistical model. The model results will be used to trigger possible adaptations. All statistical triggers are evaluated using posterior probabilities related to the odds-ratio (OR) comparing the investigational intervention to the reference intervention within a "cell" where a cell is a combination of domain and silo. An OR < 1 corresponds to a reduction in mortality.

Early stopping rules are based on the posterior probabilities for the adult age group, though these quantities are informed by the child age group due to the Bayesian hierarchical model. Decision rule thresholds are not specifically defined for the child age group.

The statistical triggers for the adult age group are defined as:

1. **Non-inferiority**:
   - An investigational intervention is determined to be non-inferior to the reference intervention if the posterior probability of the odds-ratio being below 1.2 is greater than 99%:
   $$\Pr(\text{OR} < 1.2) > 0.99.$$
   - If the reference intervention had a mortality rate of 15%, then OR = 1.2 would correspond to an absolute increase in mortality of 2.5% for the investigational intervention.
   - Non-inferiority is only assessed for Domain D_3 (early oral stepdown) and for the PSSA and MSSA silos ($s = 1, 2$) in Domain D_1 (backbone antibiotic). If non-inferiority is met for the PSSA or MSSA silos in Domain D_1, randomization may continue in these cells following the non-inferiority trigger so that superiority may be evaluated.
2. **Superiority**:
   - An investigational intervention is determined to be superior to the reference intervention if the posterior probability of the odds-ratio being below 1 is greater than 99%:
   $$\Pr(\text{OR} < 1.0) > 0.99.$$
   - In the PSSA and MSSA silos for Domain D_1, the superiority test is only considered once non-inferiority has been met.
   - Superiority is not assessed in Domain D_3 (early oral stepdown).
   - In Domain D_2 (adjunctive antibiotic), there is a single estimate of the treatment effect pooled across all silos. Thus there is a single test for superiority (not separate tests per silo) in this domain.
3. **Futility for the non-inferiority test**:
   - The test for non-inferiority is considered futile if there is less than a 1% posterior probability of the odds-ratio being below 1.2:
   $$\Pr(\text{OR} < 1.2) < 0.01.$$

- Futility for the non-inferiority test is only assessed for Domain D_3 and for the PSSA and MSSA silos in Domain D_1.

4. **Futility for the superiority test**:

- The test for superiority is considered futile if there is less than a 1% posterior probability of the odds-ratio being below 1/1.2:

$$\Pr(\text{OR} < 1/1.2) < 0.01.$$

- For cells that test non-inferiority, futility for the superiority test is only considered once non-inferiority has been met.

Table 2 summarizes the statistical triggers that are evaluated for each cell (domain and silo).

Table 2: Summary of statistical triggers to be evaluated for each domain and silo

Silo	Domain D_1 (Backbone Antibiotic)	Domain D_2 (Adjunctive Antibiotic)	Domain D_3 (Early Oral Stepdown)
PSSA ($s = 1$)	Non-inferiority *Then if met...* Superiority	Superiority	Non-inferiority
MSSA ($s = 2$)	Non-inferiority *Then if met...* Superiority	Superiority	Non-inferiority
MRSA ($s = 3$)	Superiority	Superiority	Non-inferiority

4 Example Trials

In this section, two example trials with simulated data are presented to illustrate the adaptive process. For the selected trials, a subset of the interims are shown, with each analysis represented by a set of graphs and tables depicting:

- The status of the platform at the analysis: the time of the interim (in years since the start of the platform), the total number of participants enrolled to the platform, and the total number with known 90-day outcomes.
- Two tables (one for adults and one for children) showing the observed data per domain, silo, and randomized intervention. Each cell is color-coded by domain: yellow for D_1 (backbone antibiotic), green for D_2 (adjunctive antibiotic), and orange for D_3 (early oral stepdown). The summaries for domain D_2 (adjunctive antibiotic) are pooled across silos. The following summaries are presented in the tables:
  - N: the number of patients randomized to the intervention,
  - n: the number of patients with a known 90-day outcome,
  - y: the number of deaths within 90 days,
  - y/n: the observed 90-day mortality rate.
- A plot of the model-estimated odds-ratios (medians and 95% credible intervals). There is one panel per domain. Within each panel, there are two lines per silo. The left (solid line) corresponds to the estimated OR for adults in the silo and the right (dotted line) corresponds to the estimated OR for children in the silo. The y-axis is on the log scale. The black dashed horizontal line is a reference for an OR = 1. The dashed grey and red lines show ORs of 1.2 and 1/1.2, respectively.

- Posterior probabilities for assessing statistical triggers. Probabilities are shown for superiority (Pr(OR < 1)), noninferiority (Pr(OR < 1.2)), and futility (Pr(OR $< 1/1.2$) = Pr(OR < 0.83)). There is one panel per domain. Within each panel, there are two numbers for each type of probability. The number on the left is for the adult age group and the number on the right is for children.
- A table tracking conclusions reached over the course of the platform. There is one cell in the table for each combination of domain, silo, and age group. For domain D_2 (adjunctive antibiotic), the decision rules are not dependent on silo, so the decision is repeated for each silo.

4.1 Example Trial 1

The first interim analysis for the SNAP platform is planned when the first 500 patients have completed 90 days of follow-up and have a known mortality outcome. Figure 1 shows a snapshot of the available data at this interim for the first example trial. In this example, the interim occurred a little over one year into the trial, at which time a total of 842 patients were randomized (724 adults and 118 children).

The two tables at the top of the figure describe the observed data for adults (top) and children (bottom), color-coded by domain. Thus, the yellow cells correspond to domain D_1 (backbone antibiotic), green cells to domain D_2 (adjunctive antibiotic), and orange cells to domain D_3 (early oral stepdown). Domain D_3 (early oral switch) had fewer total patients, because only a fraction of total platform participants met eligibility requirements for this domain and had the randomized assignment revealed.

In the adjunctive antibiotic domain, 362 adults were randomized to each intervention (pooled across silos). Of the 219 patients with known outcomes in the No Clindamycin intervention, there were 30 deaths (13.7%). Of the 218 patients with known outcomes in the Clindamycin intervention, there were 24 deaths (11.0%).

The model-estimated odds ratios are presented in the middle of the figure — solid line for the adult age group and dotted line for the pediatric age group. For adults in the adjunctive antibiotic domain, the estimated OR was below 1 but the interval extended above 1. As shown in the panel below, the posterior probability of OR < 1 was 0.89, which did not meet the 0.99 threshold for superiority. Likewise, the posterior probability of OR $< 1/1.2$ was 0.705, which did not meet the futility threshold of 0.01.

At this interim, no statistical triggers were met in any domain for any silo and the platform continues to the next planned interim.

Look # 1: Year 1.1 (842 enrolled; 500 complete)

Observed data: adult

	PSSA		MSSA		MRSA		PSSA/MSSA/MRSA		PSSA		MSSA		MRSA	
	Fluc	Pen	Fluc	Cef	Vanc	Vanc+Cef	No Clind	Clind	IV	EOS	IV	EOS	IV	EOS
N	59	59	229	229	74	74	362	362	29	32	134	124	43	42
n	36	36	139	139	43	44	219	218	19	18	79	80	24	23
deaths (y)	5	4	15	16	8	6	30	24	3	3	7	9	1	3
rate (y/n)	0.139	0.111	0.108	0.115	0.186	0.136	0.137	0.110	0.158	0.167	0.089	0.113	0.042	0.130

Observed data: child

	PSSA		MSSA		MRSA		PSSA/MSSA/MRSA		PSSA		MSSA		MRSA	
	Fluc	Pen	Fluc	Cef	Vanc	Vanc+Cef	No Clind	Clind	IV	EOS	IV	EOS	IV	EOS
N	9	9	37	37	13	13	59	59	7	8	34	33	13	11
n	6	5	19	18	8	7	33	30	5	5	18	16	7	6
deaths (y)	0	0	1	0	0	0	1	0	0	0	0	1	0	0
rate (y/n)	0.000	0.000	0.053	0.000	0.000	0.000	0.030	0.000	0.000	0.000	0.000	0.062	0.000	0.000

Model−estimated Odds−Ratios (log scale)

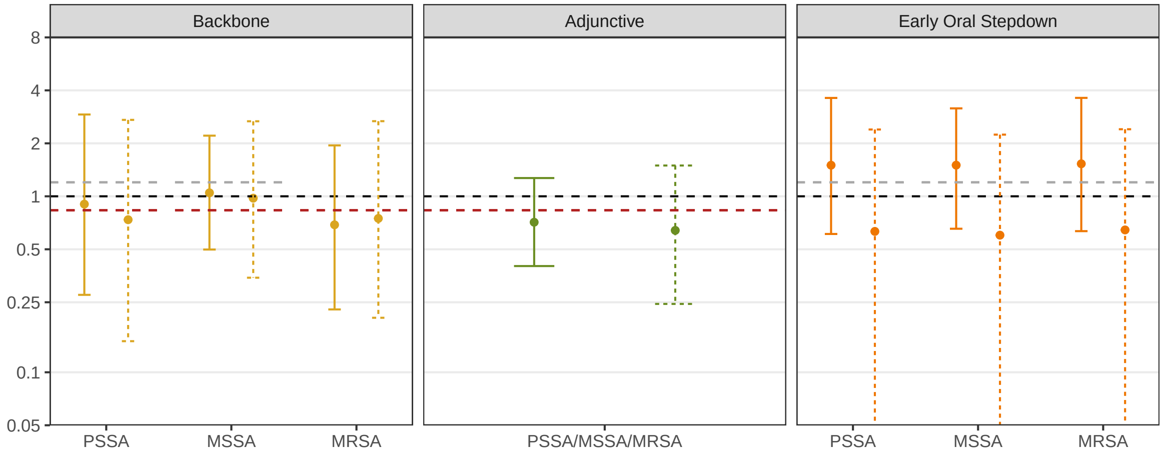

Posterior Probablities

	Backbone PSSA		Backbone MSSA		Backbone MRSA		Adjunctive PSSA/MSSA/MRSA		Early Oral Stepdown PSSA		Early Oral Stepdown MSSA		Early Oral Stepdown MRSA	
Superiority Pr(OR < 1)	0.570	0.663	0.447	0.520	0.756	0.674	0.890	0.861	0.217	0.683	0.195	0.704	0.192	0.685
Noninferiority Pr(OR < 1.2)	0.679	0.746	0.649	0.672					0.334	0.760	0.326	0.776	0.316	0.756
Futility Pr(OR < 0.83)	0.445	0.558	0.259	0.368	0.647	0.564	0.705	0.742						

Decisions

	Adult	Child	Adult	Child	Adult	Child
PSSA	No Conclusion	No Conclusion	No Conclusion	No Conclusion	No Conclusion	No Conclusion
MSSA	No Conclusion	No Conclusion	No Conclusion	No Conclusion	No Conclusion	No Conclusion
MRSA	No Conclusion	No Conclusion	No Conclusion	No Conclusion	No Conclusion	No Conclusion

Figure 1: Example Trial 1, Interim 1

Figure 2 jumps ahead to the fifth interim that occurred when 2500 patients had known 90-day mortality outcomes. For this example trial, this interim occurred 2.2 years into the trial with 3065 total patients randomized.

At this interim, a non-inferiority trigger was met for adults in domain D_1 (backbone antibiotic) within the MSSA silo. The observed mortality rates were 13.2% for flucloxacillin and 10.9% for cefazolin. The probability of noninferiority was 0.996, which met the noninferiority trigger. Randomization in this domain continues for patients in this silo so that superiority can be evaluated.

Look # 5: Year 2.2 (3065 enrolled; 2500 complete)

Observed data: adult

	PSSA		MSSA		MRSA		PSSA/MSSA/MRSA		PSSA		MSSA		MRSA	
	Fluc	Pen	Fluc	Cef	Vanc	Vanc+Cef	No Clind	Clind	IV	EOS	IV	EOS	IV	EOS
N	233	234	811	811	261	261	1306	1305	123	142	464	435	146	141
n	185	186	669	668	211	212	1065	1066	94	111	382	365	119	113
deaths (y)	18	22	88	73	49	35	157	128	10	9	34	35	20	18
rate (y/n)	0.097	0.118	0.132	0.109	0.232	0.165	0.147	0.120	0.106	0.081	0.089	0.096	0.168	0.159

Observed data: child

	PSSA		MSSA		MRSA		PSSA/MSSA/MRSA		PSSA		MSSA		MRSA	
	Fluc	Pen	Fluc	Cef	Vanc	Vanc+Cef	No Clind	Clind	IV	EOS	IV	EOS	IV	EOS
N	34	34	150	151	42	43	227	227	31	30	131	131	40	36
n	28	28	119	120	37	37	184	185	25	25	104	108	35	32
deaths (y)	1	1	1	0	0	1	3	1	1	0	0	1	1	0
rate (y/n)	0.036	0.036	0.008	0.000	0.000	0.027	0.016	0.005	0.040	0.000	0.000	0.009	0.029	0.000

Model−estimated Odds−Ratios (log scale)

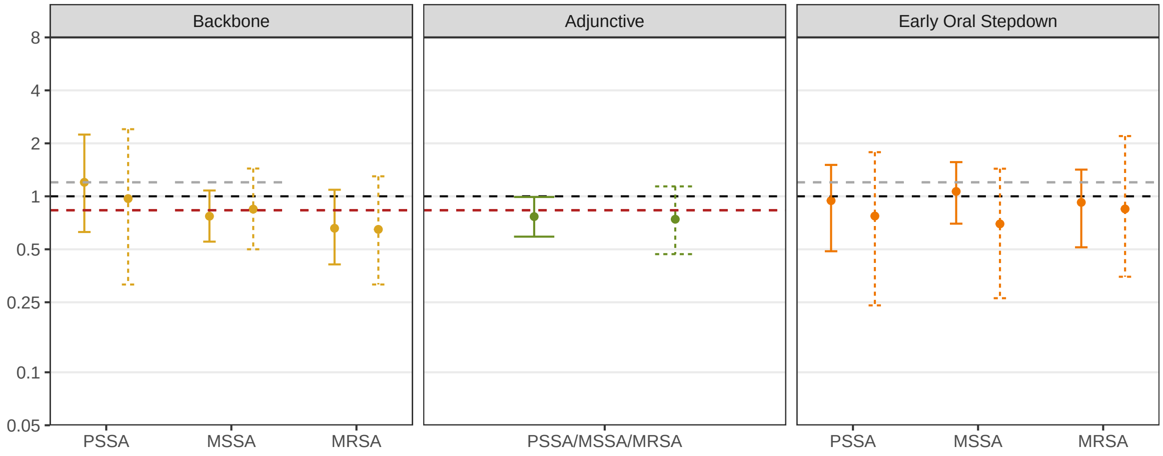

Posterior Probablities

	Backbone PSSA		Backbone MSSA		Backbone MRSA		Adjunctive PSSA/MSSA/MRSA		Early Oral Stepdown PSSA		Early Oral Stepdown MSSA		Early Oral Stepdown MRSA	
Superiority Pr(OR < 1)	0.283	0.523	0.942	0.737	0.958	0.894	0.979	0.918	0.580	0.744	0.383	0.836	0.624	0.662
Noninferiority Pr(OR < 1.2)	0.498	0.676	0.996	0.908					0.840	0.874	0.732	0.934	0.877	0.799
Futility Pr(OR < 0.83)	0.137	0.377	0.680	0.479	0.834	0.763	0.748	0.704						

Decisions

	Adult	Child	Adult	Child	Adult	Child
PSSA	No Conclusion	No Conclusion	No Conclusion	No Conclusion	No Conclusion	No Conclusion
MSSA	Non−inferiority	No Conclusion	No Conclusion	No Conclusion	No Conclusion	No Conclusion
MRSA	No Conclusion	No Conclusion	No Conclusion	No Conclusion	No Conclusion	No Conclusion

Figure 2: Example Trial 1, Interim 5

Figure 3 skips forward to the eleventh interim analysis 3.5 years into the trial. With 5500 patients having known 90-day mortality outcomes, a superiority trigger was met for domain D_2 (adjunctive antibiotic) for adults. The observed mortality rates in the domain were 13.9% for no clindamycin versus 11.9% for clindamycin. In the pediatric age group, only 8 total mortality events were observed overall in the domain, with 8 of them occurring in the no clindamycin intervention. The posterior probabilities of an odds ratio less than 1 were 0.992 for adults and 0.938 for children. Randomization is discontinued in this domain for both adults and children.

Look # 11: Year 3.5 (6019 enrolled; 5500 complete)

Observed data: adult

	PSSA		MSSA		MRSA		PSSA/MSSA/MRSA		PSSA		MSSA		MRSA	
	Fluc	Pen	Fluc	Cef	Vanc	Vanc+Cef	No Clind	Clind	IV	EOS	IV	EOS	IV	EOS
N	425	426	1630	1630	519	519	2575	2574	228	243	915	863	289	301
n	395	396	1484	1484	472	473	2353	2351	212	229	826	787	268	276
deaths (y)	44	40	186	175	85	76	326	280	25	21	79	72	37	41
rate (y/n)	0.111	0.101	0.125	0.118	0.180	0.161	0.139	0.119	0.118	0.092	0.096	0.091	0.138	0.149

Observed data: child

	PSSA		MSSA		MRSA		PSSA/MSSA/MRSA		PSSA		MSSA		MRSA	
	Fluc	Pen	Fluc	Cef	Vanc	Vanc+Cef	No Clind	Clind	IV	EOS	IV	EOS	IV	EOS
N	70	70	284	284	81	81	435	435	62	63	253	255	74	70
n	65	64	257	258	76	76	397	399	59	58	228	230	70	66
deaths (y)	1	1	4	1	0	2	8	1	1	0	1	3	1	0
rate (y/n)	0.015	0.016	0.016	0.004	0.000	0.026	0.020	0.003	0.017	0.000	0.004	0.013	0.014	0.000

Model−estimated Odds−Ratios (log scale)

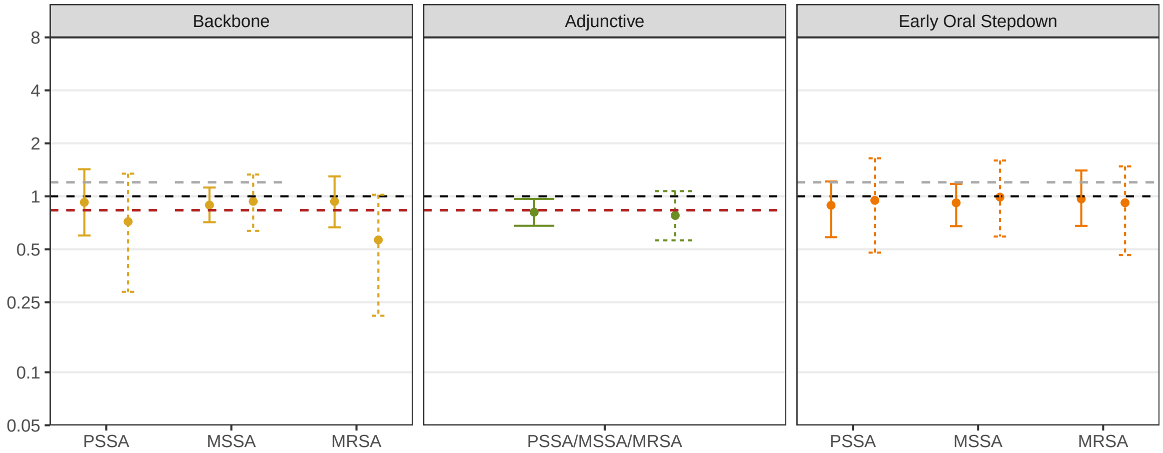

Posterior Probablities

	Backbone						Adjunctive		Early Oral Stepdown					
	PSSA		MSSA		MRSA		PSSA/MSSA/MRSA		PSSA		MSSA		MRSA	
Superiority Pr(OR < 1)	0.650	0.841	0.839	0.636	0.653	0.970	0.992	0.938	0.764	0.575	0.731	0.515	0.584	0.622
Noninferiority Pr(OR < 1.2)	0.881	0.942	0.992	0.913					0.970	0.804	0.983	0.776	0.895	0.850
Futility Pr(OR < 0.83)	0.317	0.664	0.276	0.275	0.230	0.879	0.608	0.652						

Decisions

	Adult	Child	Adult	Child	Adult	Child
PSSA	No Conclusion	No Conclusion	Stop randomizing Superiority	Stop randomizing No Conclusion	No Conclusion	No Conclusion
MSSA	Non−inferiority	No Conclusion	Stop randomizing Superiority	Stop randomizing No Conclusion	No Conclusion	No Conclusion
MRSA	No Conclusion	No Conclusion	Stop randomizing Superiority	Stop randomizing No Conclusion	No Conclusion	No Conclusion

Figure 3: Example Trial 1, Interim 11

The final analysis for this example trial is shown in Figure 4, after 7000 total patients were enrolled and followed for 90 days. At this analysis two triggers for noninferiority were met in domain D_3 (early oral stepdown) in the PSSA and MSSA silos. The observed mortality rates for these to silos were slightly lower in the early oral stepdown intervention compared to the continued IV intervention. In the MRSA silo, the observed data was very similar between interventions, and while the hierarchical model across silos did slightly shift the estimated OR for the MRSA silo because of the positive data in the other silos, the posterior probability remained below the threshold for a trigger. Very few events were observed in this domain for the pediatric age group, and the model estimated odds ratios were centered around 1 for all three silos.

In the backbone domain, the MSSA silo, which had triggered non-inferiority at a prior interim, never hit the trigger for superiority, and in fact the observed mortality rates were about equal in the two interventions. Similarly, the mortality rates in the PSSA silo were about equal, but with the smaller sample size than MSSA, the credible intervals for the OR in this silo were wider, and the noninferiority trigger was never reached.

In summary, this example trial randomized 7000 patients (5984 adults and 1016 children). Over the course of 4.2 years, 4 statistical triggers were met across the three domains.

Look # 14: Year 4.2 (7000 enrolled; 7000 complete)

Observed data: adult

	PSSA		MSSA		MRSA		PSSA/MSSA/MRSA		PSSA		MSSA		MRSA	
	Fluc	Pen	Fluc	Cef	Vanc	Vanc+Cef	No Clind	Clind	IV	EOS	IV	EOS	IV	EOS
N	501	501	1888	1887	604	603	2784	3200	269	287	1054	1008	341	352
n	501	501	1888	1887	604	603	2784	3200	269	287	1054	1008	341	352
deaths (y)	62	60	231	222	112	100	405	382	33	30	106	82	49	49
rate (y/n)	0.124	0.120	0.122	0.118	0.185	0.166	0.145	0.119	0.123	0.105	0.101	0.081	0.144	0.139

Observed data: child

	PSSA		MSSA		MRSA		PSSA/MSSA/MRSA		PSSA		MSSA		MRSA	
	Fluc	Pen	Fluc	Cef	Vanc	Vanc+Cef	No Clind	Clind	IV	EOS	IV	EOS	IV	EOS
N	82	82	330	330	96	96	471	545	71	73	294	295	85	82
n	82	82	330	330	96	96	471	545	71	73	294	295	85	82
deaths (y)	1	1	5	3	1	2	10	3	1	0	1	3	1	1
rate (y/n)	0.012	0.012	0.015	0.009	0.010	0.021	0.021	0.006	0.014	0.000	0.003	0.010	0.012	0.012

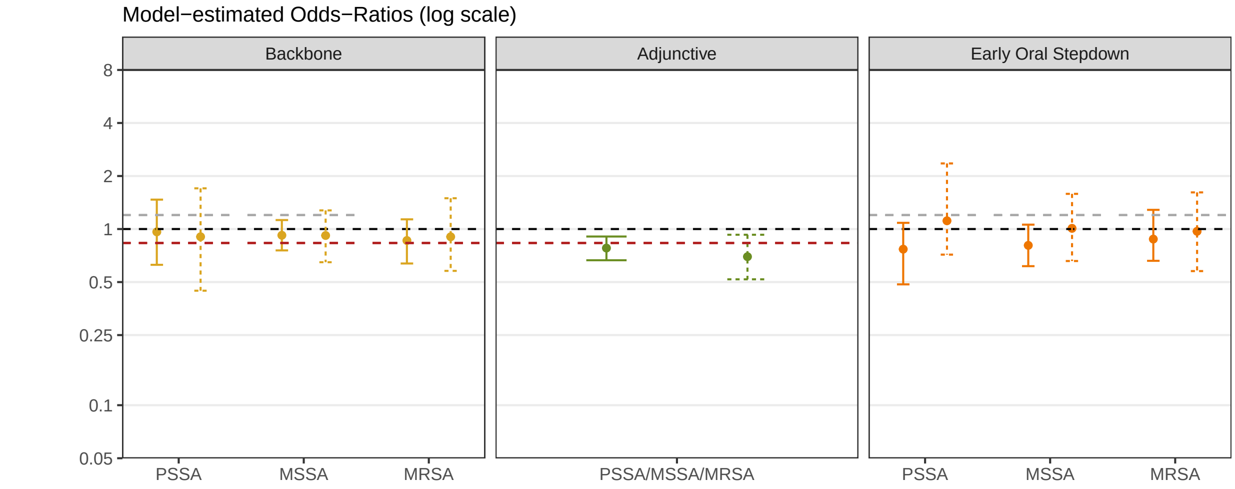

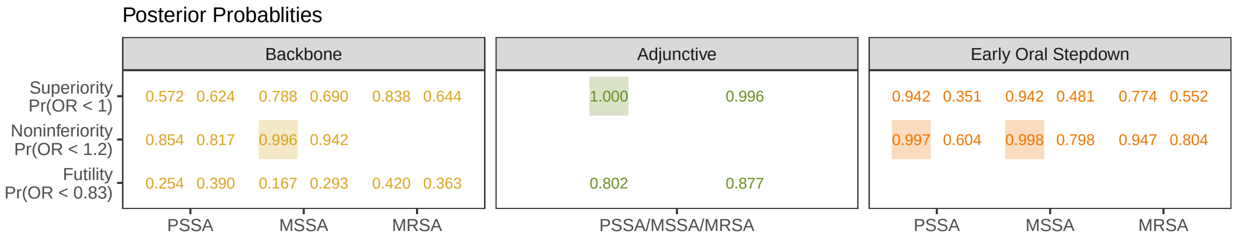

Decisions

	Adult	Child	Adult	Child	Adult	Child
PSSA	No Conclusion	No Conclusion	Stop randomizing Superiority	Stop randomizing No Conclusion	Stop randomizing Non−inferiority	Stop randomizing No Conclusion
MSSA	Non−inferiority	No Conclusion	Stop randomizing Superiority	Stop randomizing No Conclusion	Stop randomizing Non−inferiority	Stop randomizing No Conclusion
MRSA	No Conclusion	No Conclusion	Stop randomizing Superiority	Stop randomizing No Conclusion	No Conclusion	No Conclusion

Figure 4: Example Trial 1, Interim 14

4.2 Example Trial 2

For this example trial based on simulated data, no statistical triggers were met until the third interim analysis, shown in Figure 5 which occurred once 1500 patients completed 90 days of follow-up and had known mortality outcomes. There were 2050 total patients randomized. Within domain D_3 (early oral stepdown), the MSSA and MRSA silos both reached triggers for noninferiority in the adult age group. In the MSSA silo, there were 29 deaths out of 214 patients (13.6%) in the IV treatment arm and 15 deaths out of 214 patient (7%) in the early oral stepdown arm. The probability of noninferiority was $\Pr(\text{OR} < 1.2) = 0.995$ which exceeded the trigger threshold.

Similarly, within the MRSA silo, there were 17 deaths out of 74 patients in the IV treatment arm and 9 deaths out of 70 patients in the early oral stepdown arm. The probability of noninferiority exceeded the trigger (rounding in the plot puts the probability at 0.990).

Despite the hierarchical model that borrows across silos in this domain, the PSSA silo did not meet the stopping trigger at this interim and thus continues to randomize between the IV and oral arms.

Very few deaths were observed in the pediatric age group. Because triggers were reached for adults in the MSSA and MRSA silos, randomization of children in those silos is discontinued. Though no specific thresholds were specified for the pediatric age group, the posterior probabilities of noninferiority are high, largely based on the hieararchical model that is informed by the data in the adult age group.

Look # 3: Year 1.7 (2050 enrolled; 1500 complete)

Observed data: adult

	PSSA		MSSA		MRSA		PSSA/MSSA/MRSA		PSSA		MSSA		MRSA	
	Fluc	Pen	Fluc	Cef	Vanc	Vanc+Cef	No Clind	Clind	IV	EOS	IV	EOS	IV	EOS
N	144	143	549	549	172	173	864	866	78	77	298	300	97	99
n	105	105	405	405	125	126	636	635	59	56	214	214	74	70
deaths (y)	15	13	51	51	24	29	97	86	7	8	29	15	17	9
rate (y/n)	0.143	0.124	0.126	0.126	0.192	0.230	0.153	0.135	0.119	0.143	0.136	0.070	0.230	0.129

Observed data: child

	PSSA		MSSA		MRSA		PSSA/MSSA/MRSA		PSSA		MSSA		MRSA	
	Fluc	Pen	Fluc	Cef	Vanc	Vanc+Cef	No Clind	Clind	IV	EOS	IV	EOS	IV	EOS
N	24	23	103	102	34	34	160	160	23	24	87	95	32	30
n	15	15	73	72	27	27	114	115	15	15	63	67	26	25
deaths (y)	0	0	1	0	0	0	1	0	0	0	0	1	0	0
rate (y/n)	0.000	0.000	0.014	0.000	0.000	0.000	0.009	0.000	0.000	0.000	0.000	0.015	0.000	0.000

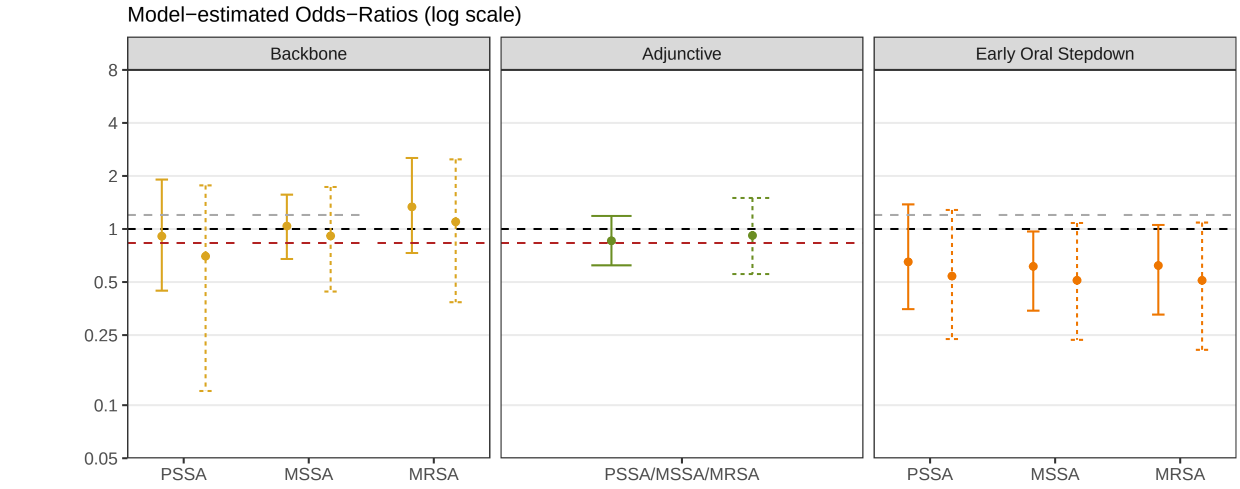

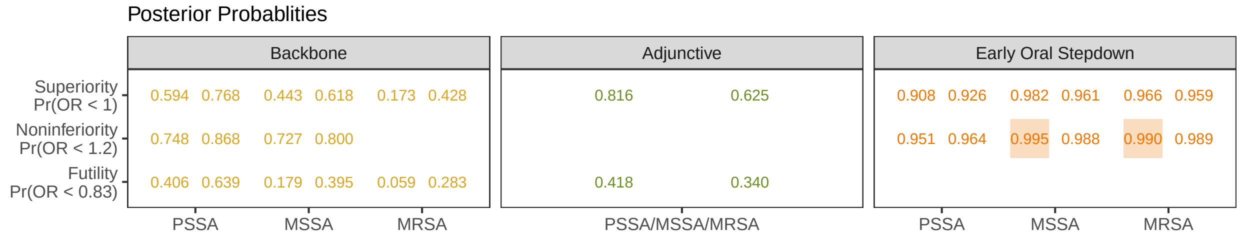

Decisions

	Adult	Child	Adult	Child	Adult	Child
PSSA	No Conclusion	No Conclusion	No Conclusion	No Conclusion	No Conclusion	No Conclusion
MSSA	No Conclusion	No Conclusion	No Conclusion	No Conclusion	Stop randomizing Non−inferiority	Stop randomizing No Conclusion
MRSA	No Conclusion	No Conclusion	No Conclusion	No Conclusion	Stop randomizing Non−inferiority	Stop randomizing No Conclusion

Figure 5: Example Trial 2, Interim 3

By the seventh interim analysis, shown in Figure 6, the PSSA silo met the criterion for non-inferiority of early oral stepdown compared to continued IV treatment. This conclusion was influenced by the hierarchical model that shares information from the MSSA and MRSA silos.

Look # 7: Year 2.6 (4040 enrolled; 3500 complete)

Observed data: adult

	PSSA		MSSA		MRSA		PSSA/MSSA/MRSA		PSSA		MSSA		MRSA	
	Fluc	Pen	Fluc	Cef	Vanc	Vanc+Cef	No Clind	Clind	IV	EOS	IV	EOS	IV	EOS
N	265	266	1106	1106	341	340	1712	1712	128	145	460	758	152	241
n	231	231	953	952	298	299	1482	1482	115	116	419	638	139	209
deaths (y)	29	27	133	133	57	58	224	213	14	15	66	65	26	32
rate (y/n)	0.126	0.117	0.140	0.140	0.191	0.194	0.151	0.144	0.122	0.129	0.158	0.102	0.187	0.153

Observed data: child

	PSSA		MSSA		MRSA		PSSA/MSSA/MRSA		PSSA		MSSA		MRSA	
	Fluc	Pen	Fluc	Cef	Vanc	Vanc+Cef	No Clind	Clind	IV	EOS	IV	EOS	IV	EOS
N	42	43	200	201	65	65	309	307	36	45	133	231	47	72
n	34	34	177	177	57	57	268	268	31	33	122	198	44	62
deaths (y)	0	0	2	2	1	0	3	2	0	0	2	1	1	0
rate (y/n)	0.000	0.000	0.011	0.011	0.018	0.000	0.011	0.007	0.000	0.000	0.016	0.005	0.023	0.000

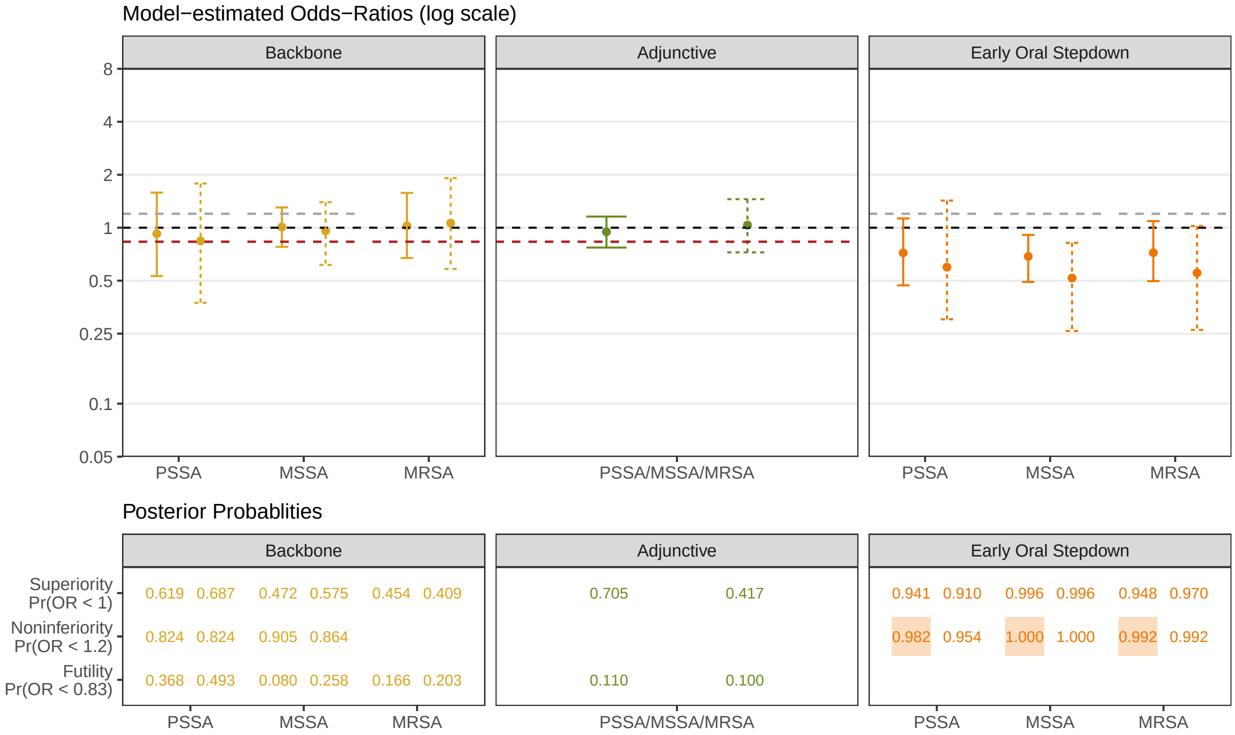

Decisions

	Adult	Child	Adult	Child	Adult	Child
PSSA	No Conclusion	No Conclusion	No Conclusion	No Conclusion	Stop randomizing Non−inferiority	Stop randomizing No Conclusion
MSSA	No Conclusion	No Conclusion	No Conclusion	No Conclusion	Stop randomizing Non−inferiority	Stop randomizing No Conclusion
MRSA	No Conclusion	No Conclusion	No Conclusion	No Conclusion	Stop randomizing Non−inferiority	Stop randomizing No Conclusion

Figure 6: Example Trial 2, Interim 7

At the 10th interim analysis, 3.3 years into the trial, there were 5000 patients with known 90-day mortality outcomes, as shown in Figure 7. Domain D_2 (adjunctive antibiotic) declared futility for clindamycin superiority over no clindamycin in adults. The estimated odds ratio was centered on 1.00 and the probability of a meaningful effect was $\Pr(\text{OR} < 1/1.2) = 0.007$ which was smaller than the futility threshold of 0.1. Randomization is discontinued in this domain for both adults and children in all silos.

Also at this interim, the MRSA silo declared futility for superiority of Vancomycin + Cefazolin in domain D_1 (backbone antibiotic). Of the 427 adults with known outcomes in the Vancomycin + Cefazolin arm, there were 95 deaths (22.2%) compared to 78 deaths out of 427 patients (18.3%) for Vancomycin alone. The probability of a meaningful effect was $\Pr(\text{OR} < 1/1.2) = 0.003$.

Look # 10: Year 3.3 (5514 enrolled; 5000 complete)

Observed data: adult

	PSSA		MSSA		MRSA		PSSA/MSSA/MRSA		PSSA		MSSA		MRSA	
	**Fluc**	**Pen**	**Fluc**	**Cef**	**Vanc**	**Vanc+Cef**	**No Clind**	**Clind**	**IV**	**EOS**	**IV**	**EOS**	**IV**	**EOS**
N	368	367	1508	1507	473	473	2348	2348	160	236	563	1078	189	349
n	335	334	1371	1370	427	427	2132	2132	149	204	530	974	178	311
deaths (y)	45	41	211	209	78	95	338	341	16	26	82	116	34	57
rate (y/n)	0.134	0.123	0.154	0.153	0.183	0.222	0.159	0.160	0.107	0.127	0.155	0.119	0.191	0.183

Observed data: child

	PSSA		MSSA		MRSA		PSSA/MSSA/MRSA		PSSA		MSSA		MRSA	
	**Fluc**	**Pen**	**Fluc**	**Cef**	**Vanc**	**Vanc+Cef**	**No Clind**	**Clind**	**IV**	**EOS**	**IV**	**EOS**	**IV**	**EOS**
N	57	57	266	265	87	86	408	410	43	66	164	321	56	99
n	52	52	239	238	77	78	369	367	40	59	151	283	53	89
deaths (y)	0	0	3	2	1	0	4	2	0	0	2	2	1	0
rate (y/n)	0.000	0.000	0.013	0.008	0.013	0.000	0.011	0.005	0.000	0.000	0.013	0.007	0.019	0.000

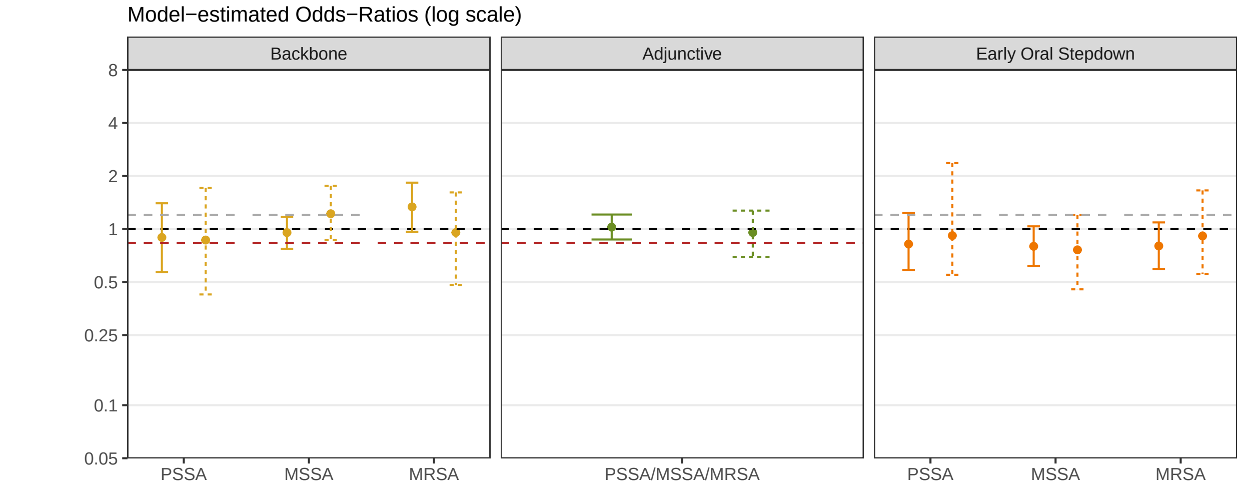

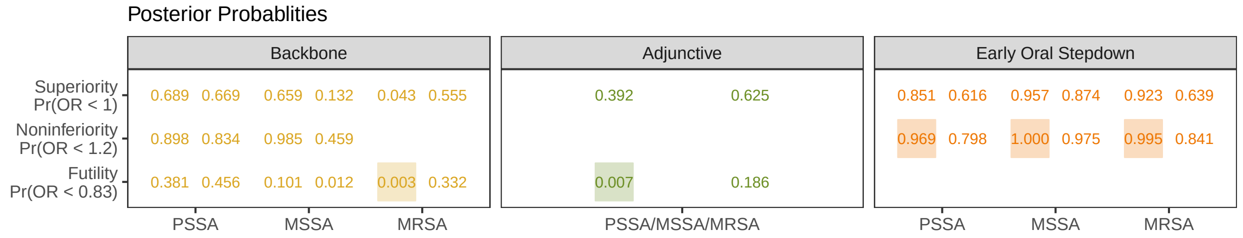

Decisions

	**Adult**	**Child**	**Adult**	**Child**	**Adult**	**Child**
PSSA	No Conclusion	No Conclusion	Stop randomizing Futility for Sup	Stop randomizing No Conclusion	Stop randomizing Non−inferiority	Stop randomizing No Conclusion
MSSA	No Conclusion	No Conclusion	Stop randomizing Futility for Sup	Stop randomizing No Conclusion	Stop randomizing Non−inferiority	Stop randomizing No Conclusion
MRSA	Stop randomizing Futility for Sup	Stop randomizing No Conclusion	Stop randomizing Futility for Sup	Stop randomizing No Conclusion	Stop randomizing Non−inferiority	Stop randomizing No Conclusion

Figure 7: Example Trial 2, Interim 10

No additional triggers were reached in this example trial until the last analysis which occurred with 7000 total patients with 90-day follow-up. As shown in Figure 8, noninferiority was triggered in the backbone antibiotic domain for the MSSA silo, with observed mortality rates of 15.3% for Cefazolin and 15.9% for flucloxacillin. The probability of noninferiority was $\Pr(\text{OR} < 1.2) = 0.992$.

Over the 4.3-year course of the platform, six statistical triggers were met across the three domains.

Look # 14: Year 4.3 (7000 enrolled; 7000 complete)

Observed data: adult

	PSSA		MSSA		MRSA		PSSA/MSSA/MRSA		PSSA		MSSA		MRSA	
	Fluc	Pen	Fluc	Cef	Vanc	Vanc+Cef	No Clind	Clind	IV	EOS	IV	EOS	IV	EOS
N	464	464	1905	1904	669	538	3283	2661	188	314	673	1401	227	456
n	464	464	1905	1904	669	538	3283	2661	188	314	673	1401	227	456
deaths (y)	60	63	303	291	127	116	534	426	20	41	109	177	44	85
rate (y/n)	0.129	0.136	0.159	0.153	0.190	0.216	0.163	0.160	0.106	0.131	0.162	0.126	0.194	0.186

Observed data: child

	PSSA		MSSA		MRSA		PSSA/MSSA/MRSA		PSSA		MSSA		MRSA	
	Fluc	Pen	Fluc	Cef	Vanc	Vanc+Cef	No Clind	Clind	IV	EOS	IV	EOS	IV	EOS
N	70	71	349	348	121	97	587	469	48	85	203	435	68	128
n	70	71	349	348	121	97	587	469	48	85	203	435	68	128
deaths (y)	0	0	4	7	2	0	9	4	0	0	3	5	2	0
rate (y/n)	0.000	0.000	0.011	0.020	0.017	0.000	0.015	0.009	0.000	0.000	0.015	0.011	0.029	0.000

Model−estimated Odds−Ratios (log scale)

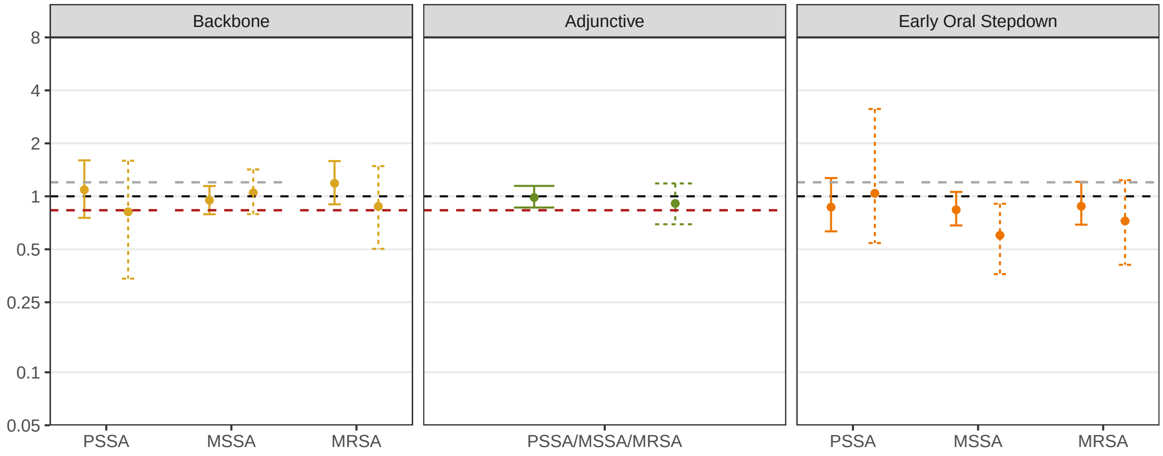

Posterior Probablities

	Backbone: PSSA		Backbone: MSSA		Backbone: MRSA		Adjunctive: PSSA/MSSA/MRSA		Early Oral Stepdown: PSSA		Early Oral Stepdown: MSSA		Early Oral Stepdown: MRSA	
Superiority Pr(OR < 1)	0.338	0.735	0.699	0.377	0.109	0.682	0.581	0.767	0.803	0.474	0.937	0.993	0.829	0.898
Noninferiority Pr(OR < 1.2)	0.697	0.876	0.992	0.810					0.958	0.601	0.999	1.000	0.972	0.970
Futility Pr(OR < 0.83)	0.076	0.520	0.076	0.055	0.009	0.429	0.008	0.256						

Decisions

	Adult	Child	Adult	Child	Adult	Child
PSSA	No Conclusion	No Conclusion	Stop randomizing Futility for Sup	Stop randomizing No Conclusion	Stop randomizing Non−inferiority	Stop randomizing No Conclusion
MSSA	Non−inferiority	No Conclusion	Stop randomizing Futility for Sup	Stop randomizing No Conclusion	Stop randomizing Non−inferiority	Stop randomizing No Conclusion
MRSA	Stop randomizing Futility for Sup	Stop randomizing No Conclusion	Stop randomizing Futility for Sup	Stop randomizing No Conclusion	Stop randomizing Non−inferiority	Stop randomizing No Conclusion

Figure 8: Example Trial 2, Interim 14

5 Simulation Scenarios

The operating characteristics of this trial were determined through trial simulation. We hypothesized several scenarios for the underlying mortality rates for each silo and age group and for the odd-ratios within each domain and silo. We then simulated the entire trial multiple times under each scenario. In each virtual trial, the interim analyses were performed according to the pre-specified rules, and results were tracked for each trial, including whether any statistical triggers were met (and in which domain and silo).

An extensive set of simulations were performed during the building of the platform trial design. The form of the statistical model, the selection of priors, and the thresholds for statistical triggers were all selected through a simulation process. In this report, we do not present the full exploration of simulations that informed the design and model decisions, but rather just present the results for the final design.

This section describes the parameters that were used to simulate the patient-level data for the virtual trials.

5.1 Baseline Patient Characteristics

For each virtual patient, the 90-day mortality outcome is simulated based on treatment allocations along with a set of baseline characteristics that include silo and age group.

Patients are classified into three groups, “silos,” based on susceptibility. For each virtual patient, the silo membership is randomly generated from probabilities:

- PSSA ($s = 1$): 16%
- MSSA ($s = 2$): 64%
- MRSA ($s = 3$): 20%

The age group category for each virtual patient is generated from a Bernoulli distribution with 85.7% probability for adult. Thus, of the 7000 patients in the trial, approximately 6000 patients, on average, will be adult. We do not simulate actual ages for the virtual patients. In the implementation of the actual trial with real data, the model will adjust for finer grain age categories, but for the simulations, the model only adjusts broadly for child versus adult.

5.2 Accrual Rate

Patient arrival times to the trial are simulated from a Poisson process. We assume that accrual begins slowly and ramps up as sites come on board. Roughly 10% of patients are expected to enroll in year 1, 25% in year 2, and full accrual is achieved, on average, in 4 years. Figure 9 shows the expected cumulative number of patients enrolled over time. Note that the graph only shows the mean expectation. Actual accrual is simulated using exponential distributions for the interval between patients. Thus, some simulated trials recruit more quickly than this average and some more slowly.

5.3 Intervention Assignments

Within each age group and silo, virtual patients are randomized in a 1:1 ratio between the active and control interventions for each domain.

5.4 Intervention Reveal

For Domains D_1 (backbone antibiotic) and D_2 (adjunctive antibiotic), every simulated patient has a randomized, revealed treatment assignment. The reveal happens immediately for these two domains in the simulations. In practice, there may be a small delay in the reveal for the backbone domain (Domain D_1)

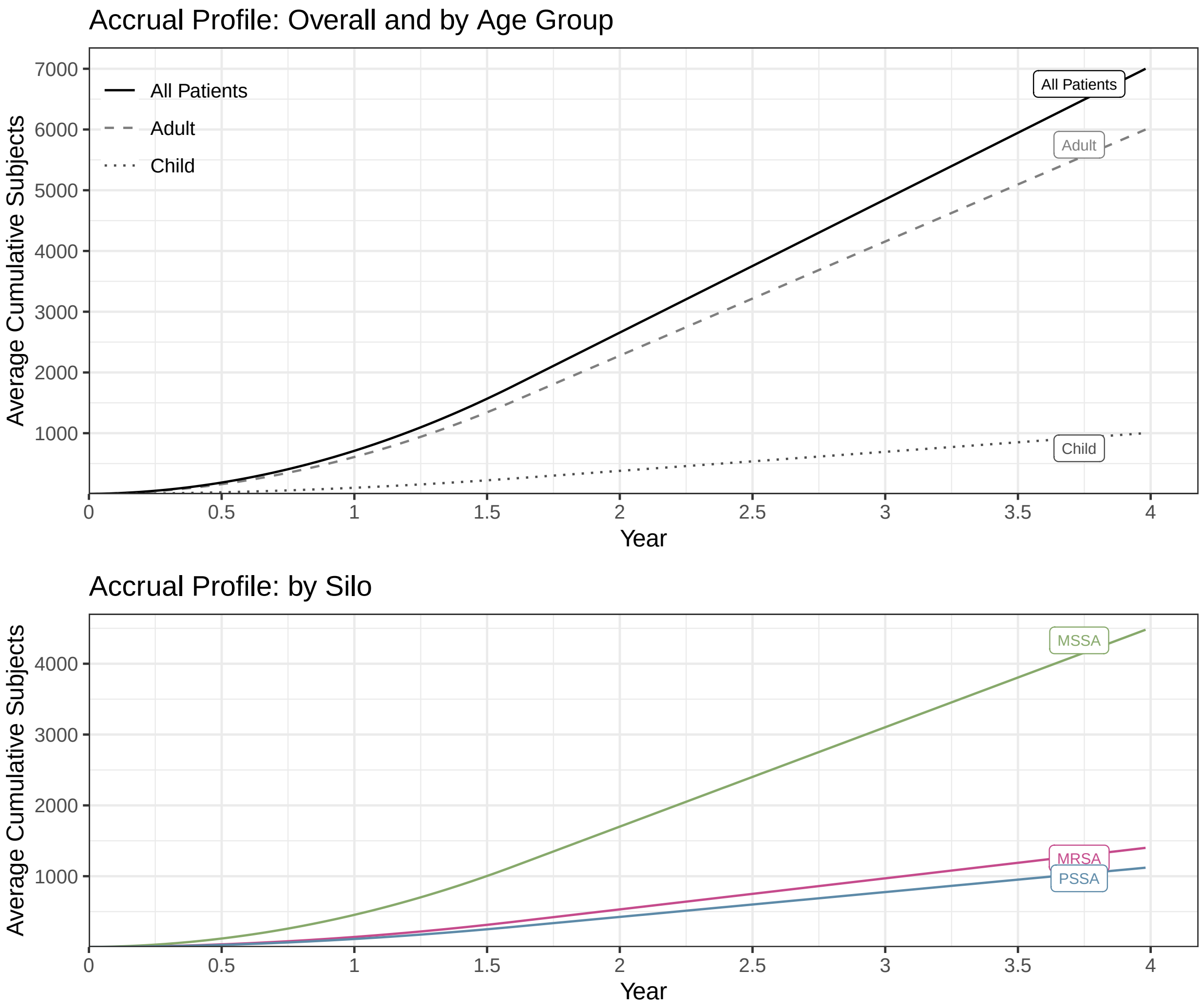

Figure 9: Accrual Profile. The top panel shows the average cumulative number of subjects over time for all patients (solid line), and by age groups (dashed line for adults and dotted line for children). The bottom panel shows the average cumulative patients (adults and children combined) within each silo.

Table 3: Percentage of patients simulated to have revealed assignment in Domain D_3 (early oral stepdown).

Reveal Category	Adults	Children
Revealed at Day 7	10%	60%
Revealed at Day 14	45%	30%
Never Revealed	45%	10%

assignment because it depends on silo, which may not be known immediately. The simulations do not explore the possibility that patients (or entire sites) may not participate in a domain.

Domain D_3 (early oral stepdown) is the only domain that is simulated to have a delayed reveal of the treatment assignment. Reveal may occur on Day 7, Day 14, or never. The percentage of patients in each reveal category differs between the adult and child age groups, as shown in Table 3.

As described in the following section, patients with a revealed allocation for Domain D_3 are simulated to have more favorable 90-day mortality outcomes (irrespective of treatment allocation) compared to those who never have the Domain D_3 allocation revealed. Likewise, patients revealed on Day 7 are simulated to have more favorable outcomes than those revealed on Day 14.

5.5 Mortality Rate Scenarios

The 90-day mortality outcome for a patient is generated from a Bernoulli distribution with rate p that depends on the patient's age group (u), silo (s), timing of eligibility for Domain D_3 ($Z_7 = 1$ if first eligible on Day 7, zero otherwise; $Z_{14} = 1$ if first eligible on Day 14, zero otherwise; both Z_7 and Z_{14} equal to zero if never eligible; it is not possible for both Z_7 and Z_{14} to be equal to one), and intervention assignments in each domain (X_1, X_2, and X_3 where $X_d = 0$ for the reference intervention and $X_d = 1$ for the investigational intervention).

$$\log\left(\frac{p}{1-p}|s, u, Z_7, Z_{14}, X_1, X_2, X_3\right) = \alpha_{s,u} + \beta_{s,u,d_{12}}X_1 + \beta_{u,d_{22}}X_2 + \beta_{s,u,d_{32}}X_3 + \gamma_7 Z_7 + \gamma_{14}Z_{14}$$

where:

- $\alpha_{s,u}$ is the log odds of the baseline mortality rate within age group u and silo s;
- $\beta_{s,u,d_{kj}}$ are the log odds-ratios for the treatment effect of the investigational intervention d_{kj} in domain d relative to the reference intervention for the silo;
- γ_7 and γ_{14} are log odds-ratios associated with being eligible for Domain D_3 (early oral stepdown) on Day 7 or Day 14, respectively, relative to never being eligible. Although the simulated mortality rates vary by whether the reveal occurs on Day 7 or Day 14, the statistical model being fit to the simulated data only adjusts for whether reveal occurs (not when it occurs).

5.5.1 Reference Mortality Rate Scenarios

Each scenario begins with a "baseline" mortality rate $\alpha_{s,u}$ that varies by silo and age group. This baseline rate is the true 90-day mortality rate for a patient assigned to the reference interventions in Domains D_1 and D_2 and with no revealed assignment in Domain D_3. The simulations assume that the baseline mortality rates are:

- For adults:
  - 0.168 for PSSA
  - 0.168 for MSSA

Table 4: Mortality scenarios for patients assigned to the reference intervention in each silo

Category	Proportion	PSSA	MSSA	MRSA
Adult				
Never revealed for Domain D_3	0.45	0.168	0.168	0.223
Reveal for Domain D_3 on Day 7	0.10	0.070	0.070	0.097
Reveal for Domain D_3 on Day 14	0.45	0.150	0.150	0.201
Overall	1.00	0.150	0.150	0.200
Child				
Never revealed for Domain D_3	0.45	0.023	0.023	0.034
Reveal for Domain D_3 on Day 7	0.10	0.009	0.009	0.013
Reveal for Domain D_3 on Day 14	0.45	0.020	0.020	0.030
Overall	1.00	0.020	0.020	0.030

  - 0.223 for MRSA

- For children:
  - 0.0227 for PSSA
  - 0.0227 for MSSA
  - 0.0345 for MRSA

These baseline mortality rates are then adjusted according to when eligibility for Domain D_3 (early oral stepdown) is determined and thus the assignment revealed. For both adults and children, the assumed odds-ratios for patients with a revealed assignment in Domain D_3 relative to no reveal are:

- 0.373 if reveal for Domain D_3 occurs on Day 7
- 0.875 if reveal for Domain D_3 occurs on Day 14

Table 4 shows the assumed mortality rates per age group and silo for patients assigned to the reference intervention in all domains, depending on when the randomized assignment was revealed for Domain D_3.

Thus, on average across the Domain D_3 reveal category, the reference mortality rates for adults are 15% in the PSSA and MSSA silos and 20% in the MRSA silo, and for children, 2% in the PSSA and MSSA silos and 4% in the MRSA silo.

5.5.2 Treatment Benefit Scenarios

We hypothesize a range of scenarios for the treatment benefit, characterized by odds-ratios.

- **Equality Scenario**: all active arms are equivalent to control (OR = 1) for all domains, all silos, and all age groups.
- **Null Scenario**: all active arms have OR = 1.2 (at the NI margin) for domains with a non-inferiority hypothesis, and OR = 1 for domains with only a superiority hypothesis.
- **Effective Scenarios**: all active arms are effective, with the same OR for all domains, all silos, and all age groups. We simulate scenarios where the OR is 0.8, 0.75, or 0.55.
- **Harm Domain D_3 Scenario**: early oral switch has higher mortality (OR = 1.5) relative to continued IV in Domain D_3 for all silos. Domains D_1 and D_2 follow the Null scenario.
- **Mixed Domain D_3 Scenarios**: Because the analysis for Domain D_3 uses a hierarchical model to borrow information across silos, we also investigate scenarios where the odds-ratio differs by silo within this domain. We consider scenarios that are perhaps implausible, but informative for the behavior of the design under extreme situations:

– The active treatment is worse than control in some silos (OR = 1.5), but equivalent in others (OR = 1). Here we primarily explore how often the design stops for futility in the poorly performing silos.
– There is treatment benefit in one or more silos (OR = 0.8), but the odds-ratio is on the non-inferiority margin in the remaining silos (OR = 1.2).

For this set of "mixed" scenarios, we assume for simplicity that the OR for other domains follow the null scenario. Table 5 shows the set of "mixed" scenarios for Domain D_3 that we explored.

Table 5: True odds-ratios for scenarios with mixed treatment effects across silos for Domain D_3

Scenario	PSSA	MSSA	MRSA
Mixed Domain D_3 01	1.0	1.0	1.5
Mixed Domain D_3 02	1.5	1.5	1.0
Mixed Domain D_3 03	1.0	1.0	1.2
Mixed Domain D_3 04	0.8	0.8	1.2
Mixed Domain D_3 05	1.2	1.2	0.8

5.5.3 Simulated outcomes following a platform conclusion

Because of the perpetual nature of the platform trial, patients will continue to be enrolled after a platform conclusion is reached in a domain. Although randomization may be discontinued within the domain, patients will continue to be randomized in other domains. Because of the multi-factorial nature of the design and the statistical model, the estimated treatment effects for each intervention in a domain adjust for the interventions in other domains. For this reason, special consideration is needed for how to approach the modeling and simulation of patients after the closure of one or more domains in a silo.

For the simulations, we make a general assumption of an average 75% clinical uptake in the recommended platform conclusion. Thus, following a conclusion in a cell (domain and silo combination), we allocate about 75% of future patients for that cell to the recommended intervention and simulate the outcome for those future patients according the true mortality rate for the set of assigned interventions.

Thus, in the simulations, allocation is changed after platform conclusions as follows:

- Superiority conclusion → change allocation to 25% reference intervention, 75% investigational intervention.
- Futility for superiority conclusion → change allocation to 75% reference intervention, 25% investigational intervention.
- Futility for non-inferiority conclusion → change allocation to 75% reference intervention, 25% investigational intervention.
- Non-inferiority conclusion for Domain D_3 → change allocation to 25% reference intervention, 75% investigational intervention. If a non-inferiority conclusion is met in Domain D_1, randomization would continue in a 1:1 ratio until a conclusion of superiority or futility for superiority was met.

In the implementation of actual trial, this deterministic allocation would not occur, but rather the patient care would be informed by clinical choice. A more sophisticated approach may be necessary for the statistical model, such as an additional covariate in the model to indicate the domain is closed and randomization no longer possible. For the simulations, we took the simplified approach described above.

6 Operating Characteristics (Adult Age Group)

For the scenarios described above, the operating characteristics of the design are demonstrated through simulation. We simulate multiple virtual trials for each scenario, conduct the design as specified above, and track the behavior of each trial, including the final outcome for each domain and silo. In this section the results are summarized for the adult age subgroup across all simulated trials for each scenario. Results for the pediatric subgroup are summarized in a later section of this report.

All results are based on 1000 virtual trials per scenario.

6.1 All Equal (OR = 1.0)

This section shows the operating characteristics in the adult age group when there is no treatment benefit (OR = 1.0) across all domains and all silos. For the superiority test, this scenario represents a null scenario. In Table 6, the probability of falsely claiming superiority in Domain D_1 (backbone antibiotic) ranges from 5% to 7% depending on silo. This is the type I error rate. For Domain D_2 (adjunctive antibiotic), the type I error rate is 7%. Remember that there is a single conclusion in Domain D_2 rather than separate conclusions per silo, but the 7% is repeated for each silo due to the table structure. There is a 69% probability of meeting the futility trigger for the superiority test in Domain D_2. On average, the futility trigger is met with an average adult sample size of 599 PSSA patients, 2392 MSSA patients, and 747 MRSA patients.

Table 6: Overall operating characteristics for Scenario: **Equality**

		Non-inferiority		Futility for the non-inferiority test		Superiority		Futility for the superiority test	
Silo	True OR	Pr(Trigger)	Mean N	Pr(Trigger)	Mean N	Pr(Trigger)	Mean N	Pr(Trigger)	Mean N
Domain D_1									
PSSA	1	0.22	706	0.00	619	0.05	599	0.00	1123
MSSA	1	0.46	2641	0.01	796	0.07	2062	0.07	3963
MRSA	1	-	-	-	-	0.06	662	0.28	809
Domain D_2									
PSSA	1	-	-	-	-	0.07	445	0.69	599
MSSA	1	-	-	-	-	0.07	1797	0.69	2392
MRSA	1	-	-	-	-	0.07	561	0.69	747
Domain D_3									
PSSA	1	0.22	688	0.00	1147	-	-	-	-
MSSA	1	0.47	2579	0.00	1180	-	-	-	-
MRSA	1	0.26	845	0.00	155	-	-	-	-

Note: Pr(Trigger) is the proportion of simulated trials (out of 1000) that met the statistical trigger. Mean N is the average sample size at the time of the statistical trigger for trials that met the trigger.

The figure shows the cumulative proportion of trials meeting each statistical trigger by interim. For example, in Domain D_2, about 50% of trials (dark red line) reached a futility trigger by the 8th interim.

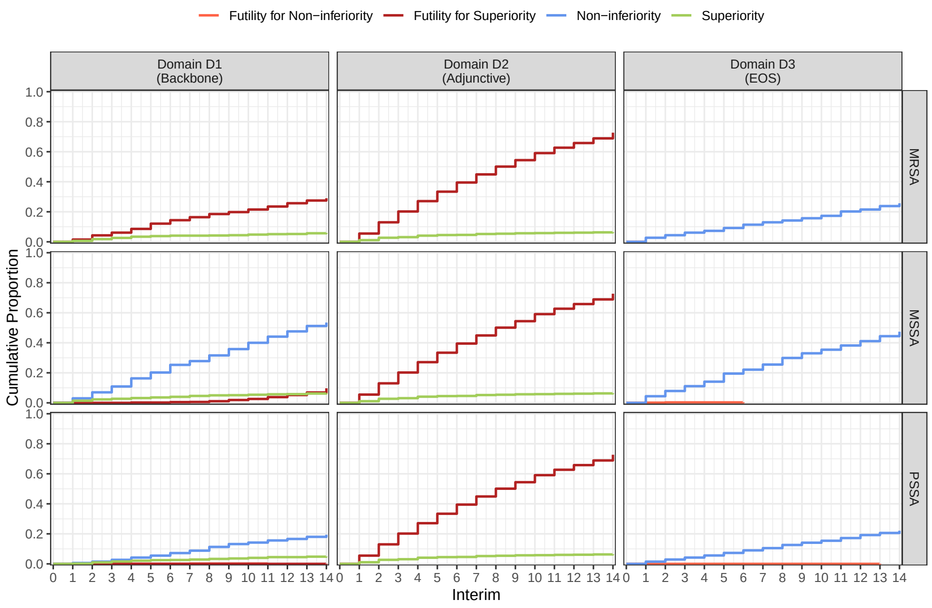

Figure 10: Triggers over time: **Equality**

6.2 All Null

In this scenario, the investigational arms all have OR = 1.2 (at the non-inferiority margin) for cells with a non-inferiority hypothesis and are equivalent to control with OR = 1.0 for cells with only a superiority hypothesis. As shown in the previous scenario, the type I error rate for the superiority hypothesis in Domain D_2 is 7%. In Domains D_1, and D_3, the type I error rate for the non-inferiority hypothesis 7% or smaller for all silos.

Table 7: Overall operating characteristics for Scenario: **Null**

		Non-inferiority		Futility for the non-inferiority test		Superiority		Futility for the superiority test	
Silo	True OR	Pr(Trigger)	Mean N	Pr(Trigger)	Mean N	Pr(Trigger)	Mean N	Pr(Trigger)	Mean N
Domain D_1									
PSSA	1.2	0.06	570	0.04	663	0.01	291	0.00	996
MSSA	1.2	0.02	2095	0.07	2041	0.00	1074	0.04	3501
MRSA	1.2	-	-	-	-	0.01	441	0.70	773
Domain D_2									
PSSA	1.0	-	-	-	-	0.07	470	0.72	587
MSSA	1.0	-	-	-	-	0.07	1880	0.72	2350
MRSA	1.0	-	-	-	-	0.07	591	0.72	735
Domain D_3									
PSSA	1.2	0.03	548	0.01	519	-	-	-	-
MSSA	1.2	0.07	1669	0.05	2156	-	-	-	-
MRSA	1.2	0.04	581	0.03	649	-	-	-	-

Note: Pr(Trigger) is the proportion of simulated trials (out of 1000) that met the statistical trigger. Mean N is the average sample size at the time of the statistical trigger for trials that met the trigger.

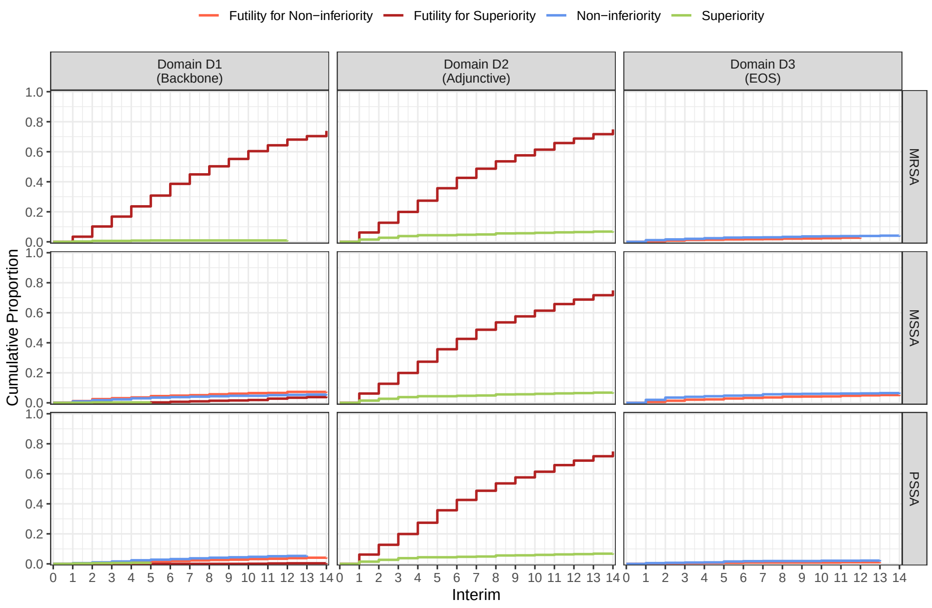

Figure 11: Triggers over time: **Null**

6.3 All Effective (OR = 0.80)

This section shows the operating characteristics in the adult age group when there is a consistent modest effect (OR = 0.80) across all domains and all silos. There is 77% power to declare superiority in Domain D_2. In Domain D_1, there is only 33% power for detecting superiority for MRSA. In silos PSSA and MSSA, the trial must first pass the non-inferiority trigger before testing superiority. The MSSA silo, being the most prevalent group, has the highest power. In Domain D_3, the power for the non-inferiority test ranges between 70% and 93% depending on silo.

Table 8: Overall operating characteristics for Scenario: **Effective (OR = 0.80)**

		Non-inferiority		Futility for the non-inferiority test		Superiority		Futility for the superiority test	
Silo	True OR	Pr(Trigger)	Mean N	Pr(Trigger)	Mean N	Pr(Trigger)	Mean N	Pr(Trigger)	Mean N
Domain D_1									
PSSA	0.8	0.51	694	0.00	358	0.21	632	0.00	-
MSSA	0.8	0.97	2027	0.00	-	0.61	2570	0.00	3221
MRSA	0.8	-	-	-	-	0.33	830	0.04	659
Domain D_2									
PSSA	0.8	-	-	-	-	0.77	587	0.04	511
MSSA	0.8	-	-	-	-	0.77	2351	0.04	2038
MRSA	0.8	-	-	-	-	0.77	735	0.04	639
Domain D_3									
PSSA	0.8	0.70	633	0.00	-	-	-	-	-
MSSA	0.8	0.93	2089	0.00	-	-	-	-	-
MRSA	0.8	0.78	744	0.00	-	-	-	-	-

Note: Pr(Trigger) is the proportion of simulated trials (out of 1000) that met the statistical trigger. Mean N is the average sample size at the time of the statistical trigger for trials that met the trigger.

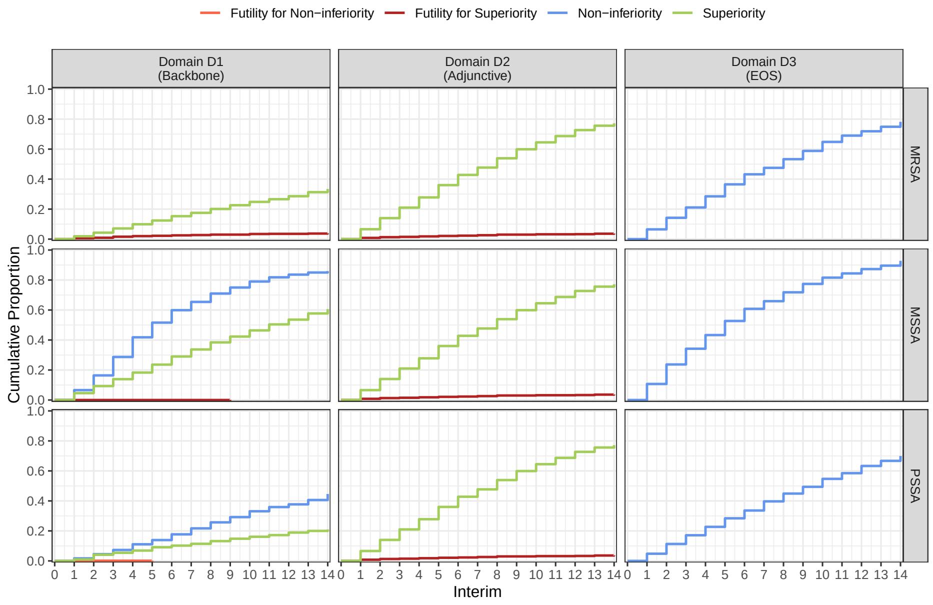

Figure 12: Triggers over time: **Effective (OR = 0.80)**

6.4 All Effective (OR = 0.75)

This scenario has a consistent moderate treatment effect (OR = 0.75) across all domains and silos. Power is above 90% for the superiority test in Domain D_2. Power for the non-inferiority test is close to 80% or higher for the non-inferiority test in Domain D_3. The PSSA silo, which is the smallest, still has only 61% power for the non-inferiority test in Domain D_1.

Table 9: Overall operating characteristics for Scenario: **Effective (OR = 0.75)**

		Non-inferiority		Futility for the non-inferiority test		Superiority		Futility for the superiority test	
Silo	True OR	Pr(Trigger)	Mean N	Pr(Trigger)	Mean N	Pr(Trigger)	Mean N	Pr(Trigger)	Mean N
Domain D_1									
PSSA	0.75	0.61	691	0.00	-	0.29	667	0.00	-
MSSA	0.75	0.99	1800	0.00	-	0.77	2439	0.00	-
MRSA	0.75	-	-	-	-	0.46	817	0.03	570
Domain D_2									
PSSA	0.75	-	-	-	-	0.93	543	0.01	305
MSSA	0.75	-	-	-	-	0.93	2175	0.01	1227
MRSA	0.75	-	-	-	-	0.93	680	0.01	386
Domain D_3									
PSSA	0.75	0.78	589	0.00	121	-	-	-	-
MSSA	0.75	0.95	1840	0.00	544	-	-	-	-
MRSA	0.75	0.84	690	0.00	-	-	-	-	-

Note: Pr(Trigger) is the proportion of simulated trials (out of 1000) that met the statistical trigger. Mean N is the average sample size at the time of the statistical trigger for trials that met the trigger.

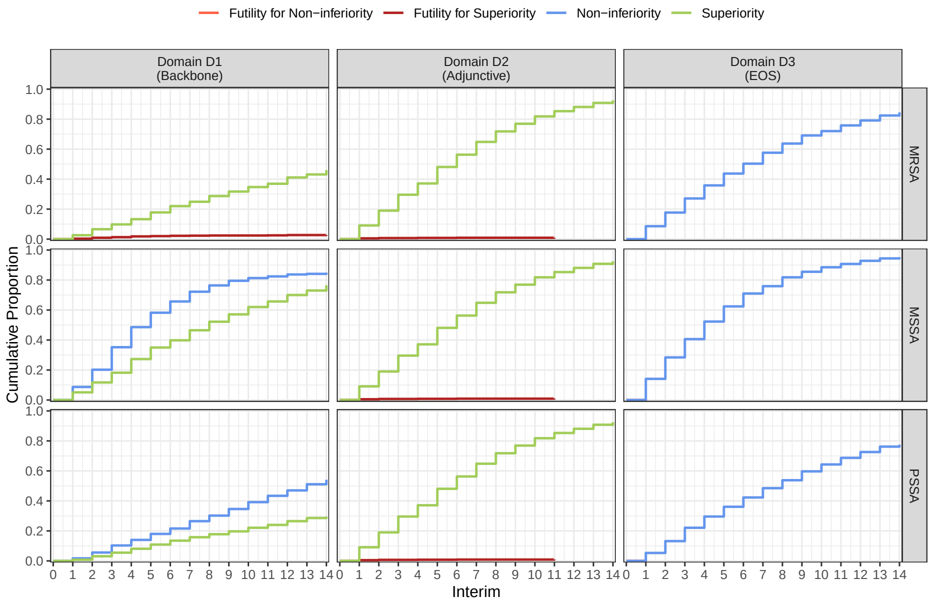

Figure 13: Triggers over time: **Effective (OR = 0.75)**

6.5 All Effective (OR = 0.55)

This scenario has a consistent large treatment effect (OR = 0.55) across all domains and silos. Power is above 80% for all of the non-inferiority tests in all domains and silos. Additionally, the average sample size at the time of trigger is smaller for this scenario than for the scenarios with OR = 0.75 and 0.80. In Domain D_2, more than 50% of trials reach the superiority trigger by the time of the 2nd interim and over 85% of trials by the 4th interim (as shown in the figure).

Table 10: Overall operating characteristics for Scenario: **Effective (OR = 0.55)**

		Non-inferiority		Futility for the non-inferiority test		Superiority		Futility for the superiority test	
Silo	True OR	Pr(Trigger)	Mean N	Pr(Trigger)	Mean N	Pr(Trigger)	Mean N	Pr(Trigger)	Mean N
Domain D_1									
PSSA	0.55	0.83	624	0.00	-	0.63	637	0.00	-
MSSA	0.55	1.00	1260	0.00	-	0.99	1615	0.00	-
MRSA	0.55	-	-	-	-	0.81	729	0.00	285
Domain D_2									
PSSA	0.55	-	-	-	-	1.00	292	0.00	155
MSSA	0.55	-	-	-	-	1.00	1166	0.00	523
MRSA	0.55	-	-	-	-	1.00	365	0.00	149
Domain D_3									
PSSA	0.55	0.95	443	0.00	-	-	-	-	-
MSSA	0.55	1.00	1297	0.00	-	-	-	-	-
MRSA	0.55	0.98	502	0.00	-	-	-	-	-

Note: Pr(Trigger) is the proportion of simulated trials (out of 1000) that met the statistical trigger. Mean N is the average sample size at the time of the statistical trigger for trials that met the trigger.

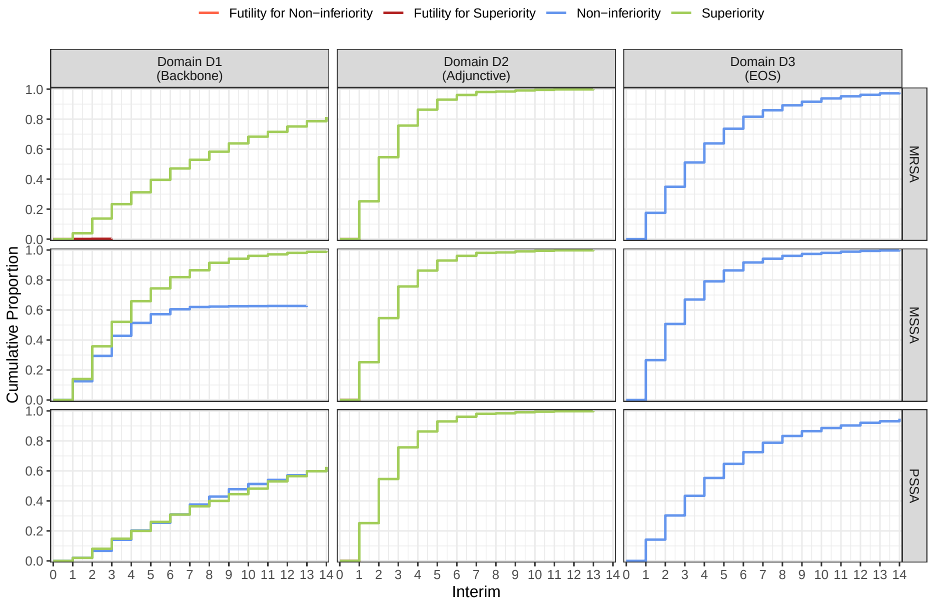

Figure 14: Triggers over time: **Effective (OR = 0.55)**

6.6 Harm Domain D_3

This scenario shows the operating characteristics if there is harm (OR = 1.5) for all silos in Domain D_3. Depending on silo, the trial meets the futility trigger for the non-inferiority test 27-57% of the time. The PSSA silo is the most difficult to stop for futility due to the small sample size within this silo. The estimated odds-ratio tends to be quite wide.

Table 11: Overall operating characteristics for Scenario: **Harm Domain** D_3

		Non-inferiority		Futility for the non-inferiority test		Superiority		Futility for the superiority test	
Silo	True OR	Pr(Trigger)	Mean N	Pr(Trigger)	Mean N	Pr(Trigger)	Mean N	Pr(Trigger)	Mean N
Domain D_1									
PSSA	1.2	0.05	542	0.05	602	0.01	410	0.00	1072
MSSA	1.2	0.02	2145	0.07	2122	0.01	676	0.05	3572
MRSA	1.2	-	-	-	-	0.01	454	0.71	786
Domain D_2									
PSSA	1.0	-	-	-	-	0.07	441	0.73	575
MSSA	1.0	-	-	-	-	0.07	1772	0.73	2295
MRSA	1.0	-	-	-	-	0.07	561	0.73	717
Domain D_3									
PSSA	1.5	0.00	-	0.27	690	-	-	-	-
MSSA	1.5	0.00	667	0.57	2654	-	-	-	-
MRSA	1.5	0.00	438	0.34	873	-	-	-	-

Note: Pr(Trigger) is the proportion of simulated trials (out of 1000) that met the statistical trigger. Mean N is the average sample size at the time of the statistical trigger for trials that met the trigger.

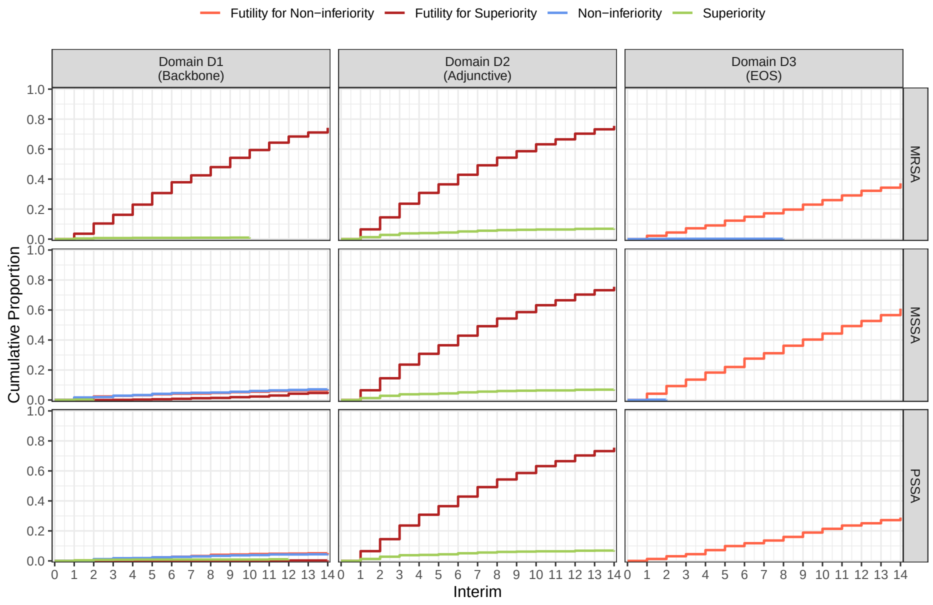

Figure 15: Triggers over time: **Harm Domain** D_3

6.7 Mixed Domain D_3

The “Mixed” scenarios for Domain D_3 are intended to illustrate the behavior of the hierarchical model in extreme scenarios that violate the anticipated similarity of effect across silos. In this first scenario, the early oral switch intervention increases the mortality rate (OR = 1.5) in the MRSA silo but has no difference (OR = 1.0) in the PSSA and MSSA silos (which is a positive scenario for the non-inferiority test). The simulations illustrate that the hierarchical borrowing between silos still maintains a low probability of a false positive test in this scenario, despite the treatment effect estimate being shrunk towards the estimates in the non-harm silos.

Table 12: Overall operating characteristics for Scenario: **Mixed Domain** D_3 **01**

		Non-inferiority		Futility for the non-inferiority test		Superiority		Futility for the superiority test	
Silo	True OR	Pr(Trigger)	Mean N	Pr(Trigger)	Mean N	Pr(Trigger)	Mean N	Pr(Trigger)	Mean N
Domain D_1									
PSSA	1.2	0.06	558	0.05	657	0.01	347	0.00	1039
MSSA	1.2	0.02	2229	0.06	1887	0.00	783	0.04	3605
MRSA	1.2	-	-	-	-	0.01	444	0.71	788
Domain D_2									
PSSA	1.0	-	-	-	-	0.08	530	0.73	583
MSSA	1.0	-	-	-	-	0.08	2139	0.73	2328
MRSA	1.0	-	-	-	-	0.08	673	0.73	727
Domain D_3									
PSSA	1.0	0.09	682	0.00	137	-	-	-	-
MSSA	1.0	0.29	2595	0.01	1616	-	-	-	-
MRSA	1.5	0.03	508	0.04	957	-	-	-	-

Note: Pr(Trigger) is the proportion of simulated trials (out of 1000) that met the statistical trigger. Mean N is the average sample size at the time of the statistical trigger for trials that met the trigger.

Table 13: Overall operating characteristics for Scenario: **Mixed Domain D_3 02**

		Non-inferiority		Futility for the non-inferiority test		Superiority		Futility for the superiority test	
Silo	True OR	Pr(Trigger)	Mean N	Pr(Trigger)	Mean N	Pr(Trigger)	Mean N	Pr(Trigger)	Mean N
Domain D_1									
PSSA	1.2	0.06	621	0.05	558	0.01	475	0.00	958
MSSA	1.2	0.01	1846	0.07	1889	0.00	659	0.04	3602
MRSA	1.2	-	-	-	-	0.01	358	0.69	776
Domain D_2									
PSSA	1.0	-	-	-	-	0.07	452	0.76	601
MSSA	1.0	-	-	-	-	0.07	1814	0.76	2406
MRSA	1.0	-	-	-	-	0.07	573	0.76	752
Domain D_3									
PSSA	1.5	0.00	372	0.12	755	-	-	-	-
MSSA	1.5	0.01	703	0.39	2723	-	-	-	-
MRSA	1.0	0.03	921	0.04	624	-	-	-	-

Note: Pr(Trigger) is the proportion of simulated trials (out of 1000) that met the statistical trigger. Mean N is the average sample size at the time of the statistical trigger for trials that met the trigger.

Table 14: Overall operating characteristics for Scenario: **Mixed Domain D_3 03**

		Non-inferiority		Futility for the non-inferiority test		Superiority		Futility for the superiority test	
Silo	True OR	Pr(Trigger)	Mean N	Pr(Trigger)	Mean N	Pr(Trigger)	Mean N	Pr(Trigger)	Mean N
Domain D_1									
PSSA	1.2	0.05	573	0.04	619	0.01	409	0.00	1076
MSSA	1.2	0.02	1794	0.07	2216	0.00	716	0.04	3423
MRSA	1.2	-	-	-	-	0.01	483	0.70	782
Domain D_2									
PSSA	1.0	-	-	-	-	0.07	496	0.73	592
MSSA	1.0	-	-	-	-	0.07	1984	0.73	2364
MRSA	1.0	-	-	-	-	0.07	622	0.73	739
Domain D_3									
PSSA	1.0	0.16	677	0.00	186	-	-	-	-
MSSA	1.0	0.39	2486	0.01	1282	-	-	-	-
MRSA	1.2	0.12	754	0.01	788	-	-	-	-

Note: Pr(Trigger) is the proportion of simulated trials (out of 1000) that met the statistical trigger. Mean N is the average sample size at the time of the statistical trigger for trials that met the trigger.

Table 15: Overall operating characteristics for Scenario: **Mixed Domain D_3 04**

Silo	True OR	Non-inferiority		Futility for the non-inferiority test		Superiority		Futility for the superiority test	
		Pr(Trigger)	Mean N	Pr(Trigger)	Mean N	Pr(Trigger)	Mean N	Pr(Trigger)	Mean N
Domain D_1									
PSSA	1.2	0.05	605	0.04	589	0.01	341	0.00	1121
MSSA	1.2	0.02	1936	0.06	1820	0.00	612	0.04	3618
MRSA	1.2	-	-	-	-	0.01	427	0.69	769
Domain D_2									
PSSA	1.0	-	-	-	-	0.07	448	0.71	584
MSSA	1.0	-	-	-	-	0.07	1796	0.71	2331
MRSA	1.0	-	-	-	-	0.07	564	0.71	728
Domain D_3									
PSSA	0.8	0.55	650	0.00	137	-	-	-	-
MSSA	0.8	0.90	2181	0.00	531	-	-	-	-
MRSA	1.2	0.28	746	0.01	858	-	-	-	-

Note: Pr(Trigger) is the proportion of simulated trials (out of 1000) that met the statistical trigger. Mean N is the average sample size at the time of the statistical trigger for trials that met the trigger.

Table 16: Overall operating characteristics for Scenario: **Mixed Domain D_3 05**

Silo	True OR	Non-inferiority		Futility for the non-inferiority test		Superiority		Futility for the superiority test	
		Pr(Trigger)	Mean N	Pr(Trigger)	Mean N	Pr(Trigger)	Mean N	Pr(Trigger)	Mean N
Domain D_1									
PSSA	1.2	0.06	613	0.04	668	0.01	339	0.00	882
MSSA	1.2	0.02	2066	0.08	1974	0.00	926	0.03	3339
MRSA	1.2	-	-	-	-	0.01	367	0.67	793
Domain D_2									
PSSA	1.0	-	-	-	-	0.08	455	0.73	582
MSSA	1.0	-	-	-	-	0.08	1833	0.73	2323
MRSA	1.0	-	-	-	-	0.08	577	0.73	725
Domain D_3									
PSSA	1.2	0.07	628	0.00	451	-	-	-	-
MSSA	1.2	0.14	2078	0.02	2164	-	-	-	-
MRSA	0.8	0.31	887	0.00	214	-	-	-	-

Note: Pr(Trigger) is the proportion of simulated trials (out of 1000) that met the statistical trigger. Mean N is the average sample size at the time of the statistical trigger for trials that met the trigger.

The type I error rate tends to be higher for the PSSA silo and lower for the MSSA silo. This is driven by the MSSA silo having a higher probability of stopping for futility, owing to having more data at each interim compared to the smaller PSSA silo.

7 Operating Characteristics (Both Age Groups)

In this section, we revisit a subset of the scenarios above, adding summaries for children to those of the adult age group. Recall that all stopping rules in the platform trial are based the posterior probability thresholds being reached for the adult age group. Once a platform conclusion is reached for adults, any public disclosure is anticipated to also report the results for children. Because of the much smaller sample size and smaller

event rate expected for children, it is unlikely that the posterior probabilities for children will meet specific thresholds at the time of an adult trigger being met. Nevertheless, this section summarizes the proportion of trials in which the pre-defined thresholds for statistical triggers (as defined for adults) would also be achieved in the child age group.

7.1 All Equal (OR = 1.0)

Table 17: Probability of statistical triggers (adults and children) for Scenario: **Equality**

	True OR		Non-inferiority		Futility for the non-inferiority test		Superiority		Futility for the superiority test	
Silo	Adult	Child	Adult	Child	Adult	Child	Adult	Child	Adult	Child
Domain D_1										
PSSA	1	1	0.22	0.02	0.00	0.00	0.05	0.01	0.00	0.00
MSSA	1	1	0.46	0.06	0.01	0.00	0.07	0.01	0.07	0.00
MRSA	1	1	-	-	-	-	0.06	0.00	0.28	0.02
Domain D_2										
PSSA	1	1	-	-	-	-	0.07	0.01	0.69	0.04
MSSA	1	1	-	-	-	-	0.07	0.01	0.69	0.04
MRSA	1	1	-	-	-	-	0.07	0.01	0.69	0.04
Domain D_3										
PSSA	1	1	0.22	0.01	0.00	0.00	-	-	-	-
MSSA	1	1	0.47	0.03	0.00	0.00	-	-	-	-
MRSA	1	1	0.26	0.01	0.00	0.00	-	-	-	-

Note: Results are based on 1000 simulated trials.

7.2 All Null

Table 18: Probability of statistical triggers (adults and children) for Scenario: **Null**

	True OR		Non-inferiority		Futility for the non-inferiority test		Superiority		Futility for the superiority test	
Silo	Adult	Child	Adult	Child	Adult	Child	Adult	Child	Adult	Child
Domain D_1										
PSSA	1.2	1.2	0.06	0.01	0.04	0.00	0.01	0.00	0.00	0.00
MSSA	1.2	1.2	0.02	0.00	0.07	0.00	0.00	0.00	0.04	0.00
MRSA	1.2	1.2	-	-	-	-	0.01	0.00	0.70	0.07
Domain D_2										
PSSA	1.0	1.0	-	-	-	-	0.07	0.00	0.72	0.05
MSSA	1.0	1.0	-	-	-	-	0.07	0.00	0.72	0.05
MRSA	1.0	1.0	-	-	-	-	0.07	0.00	0.72	0.05
Domain D_3										
PSSA	1.2	1.2	0.03	0.00	0.01	0.00	-	-	-	-
MSSA	1.2	1.2	0.07	0.01	0.05	0.00	-	-	-	-
MRSA	1.2	1.2	0.04	0.00	0.03	0.00	-	-	-	-

Note: Results are based on 1000 simulated trials.

7.3 All Effective (OR = 0.80)

Table 19: Probability of statistical triggers (adults and children) for Scenario: **Effective (OR = 0.80)**

	True OR		Non-inferiority		Futility for the non-inferiority test		Superiority		Futility for the superiority test	
Silo	Adult	Child	Adult	Child	Adult	Child	Adult	Child	Adult	Child
Domain D_1										
PSSA	0.8	0.8	0.51	0.07	0.00	0.00	0.21	0.01	0.00	0.00
MSSA	0.8	0.8	0.97	0.23	0.00	0.00	0.61	0.06	0.00	0.00
MRSA	0.8	0.8	-	-	-	-	0.33	0.03	0.04	0.00
Domain D_2										
PSSA	0.8	0.8	-	-	-	-	0.77	0.07	0.04	0.00
MSSA	0.8	0.8	-	-	-	-	0.77	0.07	0.04	0.00
MRSA	0.8	0.8	-	-	-	-	0.77	0.07	0.04	0.00
Domain D_3										
PSSA	0.8	0.8	0.70	0.04	0.00	0.00	-	-	-	-
MSSA	0.8	0.8	0.93	0.11	0.00	0.00	-	-	-	-
MRSA	0.8	0.8	0.78	0.05	0.00	0.00	-	-	-	-

Note: Results are based on 1000 simulated trials.

7.4 All Effective (OR = 0.55)

Table 20: Probability of statistical triggers (adults and children) for Scenario: **Effective (OR = 0.55)**

	True OR		Non-inferiority		Futility for the non-inferiority test		Superiority		Futility for the superiority test	
Silo	Adult	Child	Adult	Child	Adult	Child	Adult	Child	Adult	Child
Domain D_1										
PSSA	0.55	0.55	0.83	0.22	0.00	0.00	0.63	0.07	0.00	0.00
MSSA	0.55	0.55	1.00	0.41	0.00	0.00	0.99	0.17	0.00	0.00
MRSA	0.55	0.55	-	-	-	-	0.81	0.12	0.00	0.00
Domain D_2										
PSSA	0.55	0.55	-	-	-	-	1.00	0.20	0.00	0.00
MSSA	0.55	0.55	-	-	-	-	1.00	0.20	0.00	0.00
MRSA	0.55	0.55	-	-	-	-	1.00	0.20	0.00	0.00
Domain D_3										
PSSA	0.55	0.55	0.95	0.10	0.00	0.00	-	-	-	-
MSSA	0.55	0.55	1.00	0.19	0.00	0.00	-	-	-	-
MRSA	0.55	0.55	0.98	0.15	0.00	0.00	-	-	-	-

Note: Results are based on 1000 simulated trials.

8 Alternative Design Simulations

This features of the SNAP adaptive platform trial were explored through simulations. This report shows the operating characteristics based on the selected features and parameters of the final design. However, simulations were performed to explore alternative settings in the design process. For example, for Domain D_3, the amount of hierarchical borrowing of information across silos was explored by simulating the trial with several different prior distributions, with the selected prior providing the preferred trade-off between increasing power through borrowing when the silos are similar and decreasing power (or increasing type I error rate) when the silos are different.

Alternative thresholds for statistical triggers were also simulated. While a lower threshold (0.975) on the posterior probability for declaring non-inferiority or superiority resulted in higher power, it also resulted in unacceptable increase in the false positive rate. Similarly, a higher threshold for the futility threshold (0.05) was simulated, but the more conservative 0.01 threshold was selected.
